# Oxygen extraction fraction mapping between normal cognition and mild cognitive impairment in older adults using quantitative BOLD

**DOI:** 10.1162/IMAG.a.1378

**Published:** 2026-09-29

**Authors:** Linh N.N. Le, Gregory J. Wheeler, Nicholas P. Blockley, Evan Fletcher, Youngkyoo Jung, Sarah E. T. Farias, Brandon Gavett, Charles DeCarli, Audrey P. Fan

**Affiliations:** Department of Biomedical Engineering, University of California, Davis, Davis, CA, United States; School of Medicine and Health Sciences, University of Nottingham, Nottingham, United Kingdom; Department of Neurology, University of California, Davis, Davis, CA, United States; Department of Radiology, University of California, Davis, Davis, CA, United States

**Keywords:** oxygen extraction fraction (OEF), quantitative BOLD (qBOLD), magnetic resonance imaging (MRI), mild cognitive impairment (MCI), Bayesian inference, oxygen metabolism

## Abstract

Oxygen extraction fraction (OEF) represents the proportion of oxygen utilized by the brain from the blood and is a sensitive biomarker in understanding the vascular interplay between oxygen supply and demand. Alterations in OEF are increasingly recognized in normal aging and the pathophysiology of many neurological disorders, including Alzheimer’s disease and cognitive impairment. Here, we aimed to assess regional OEF MRI in older adults and investigate its relationship with mild cognitive impairment (MCI) and normal aging using a Variational Bayesian quantitative BOLD (qBOLD) framework. The qBOLD model leverages a biophysical relationship between OEF, deoxygenated blood volume (DBV), and the reversible transverse relaxation rate (R_2_^’^) to extract parameter values from asymmetric spin echo (ASE) MRI scans. The reliability of these parameter estimates was enhanced by Variational Bayesian inference, which incorporates prior distributions that we optimized in numerical simulation. We then applied this model to a cohort of older adults (N = 70) from the Alzheimer’s Disease Research Center (ADRC) at the University of California, Davis, including 58 cognitively normal (CN) and 12 amnestic mild cognitive impaired (MCI) participants. Our findings revealed a trend of elevated OEF across many cortical brain regions in the MCI group. Additionally, we identified regional positive associations between OEF and age in the MCI group, significantly in the left inferior parietal region (β=0.88±0.23, T=3.75, P=0.0004 ). Furthermore, the relationship between OEF and episodic memory in the right parahippocampal region differed significantly between two diagnostic groups (negative for CN and positive for MCI) (β=0.09±0.02, T=3.77, P=0.0004 ). This work adopts a novel qBOLD MRI framework for studying OEF changes in cognitive impairment as a potential biomarker for monitoring pathophysiology in aging and dementia.

## 1 Introduction

Oxygen extraction fraction (OEF), which measures the proportion of oxygen removed from the blood by the brain, has been under extensive research in clinical practice for evaluating tissue viability in tumor and stroke (Fan, Khalil, et al., 2020; Ito et al., 1982; W. Lin & Powers, 2018) and is increasingly recognized as a critical biomarker in the study of aging and cognitive impairment (Iadecola, 2004; Jiang et al., 2020; Thomas et al., 2017; Yang et al., 2023). Previous studies suggest that cerebral blood flow decreases with age due to vascular dysfunction, leading to a compensatory change in OEF to maintain adequate oxygenation (Jordan & DeBaun, 2018; McFadden et al., 2025). However, in conditions of cognitive impairment, such as mild cognitive impairment (MCI) and Alzheimer’s disease, the brain’s compensatory mechanisms may prove inadequate. In Alzheimer’s disease specifically, metabolic demand in brain tissue declines due to amyloidosis, tauopathy, and neurodegeneration (Edison et al., 2007), while vascular function, including cerebrovascular reactivity, remains relatively preserved (Thomas et al., 2017). These alterations in OEF have been linked to neurodegenerative processes and cognitive decline, underscoring its potential role in the pathophysiology of aging-related cognitive disorders. Understanding the alterations in OEF could provide valuable metabolic insights into the early detection and progression of cognitive impairment in the older population and ultimately support new interventions.

Brain OEF is traditionally measured using ^15^O positron emission tomography (^15^O PET), which is widely known as the gold standard to measure OEF, cerebral blood flow (CBF), and cerebral metabolic rate of oxygen consumption (CMRO_2_) (Ishii et al., 1996). Previous PET studies have shown metabolic changes associated with aging, dementia, and Alzheimer’s disease (Ishii et al., 1996; Nagata et al., 2000; Yamaguchi et al., 1986). However, the complicated process of using the isotopic radiotracer ^15^O, which has a short half-life of 2 min, causes a limitation in broader clinical use (Jiang & Lu, 2022). Recent advancements in MRI-based techniques have allowed noninvasive estimation of OEF without ionizing radiation and have been applied to investigate cerebral oxygen metabolism in aging and cognitive impairment. For example, studies using TRUST MRI have reported age-related alterations in global oxygenation from T_2_ relaxation in the sagittal sinus in older adults (Catchlove et al., 2018; Jiang et al., 2023). Jiang et al. (2020) and Thomas et al. (2017) reported a reduction in global OEF among individuals with MCI with minimal vascular risk factors, but an increase in OEF with age-related vascular disease (Jiang et al., 2020). Despite these initial observations, most prior MRI studies in aging cohorts have focused primarily on whole-brain OEF measurements, leaving regional variations in oxygen metabolism in MCI insufficiently understood. Recent MRI studies have instead used quantitative susceptibility mapping (QSM) approaches, including QSM-qBOLD techniques, to detect regional OEF alterations related to aging and cognitive impairment (Chiang et al., 2022; D. Wu et al., 2024). The studies highlight the potential of MRI to provide OEF mapping and motivate the need for robust techniques for regional OEF assessment in older cohorts.

As a promising method, the quantitative BOLD (qBOLD) model (Yablonskiy & Haacke, 1994) was developed to non-invasively construct quantitative maps of parameters related to brain metabolism and vascular health. This model was originally applied to the gradient echo sampling of spin echo (GESSE) sequence to analyze deoxyhemoglobin effects on the transverse MR signal (He & Yablonskiy, 2007). This qBOLD approach considers both extravascular and intravascular MRI signal contributions to fit for reversible transverse relaxation rate (R_2_’) and deoxygenated blood volume (DBV), and subsequently quantify OEF at the voxel level (An & Lin, 2000, 2003; He & Yablonskiy, 2007; Yablonskiy et al., 2013; Yin et al., 2018). qBOLD has been widely used for estimating OEF, particularly in studies of cerebrovascular disease (An et al., 2015; J.-M. Lee et al., 2003; Wang et al., 2021; Yin et al., 2018; Yu et al., 2013). Previous studies have also utilized qBOLD mapping methods to investigate cerebral oxygen metabolism and cerebrovascular function in healthy individuals and pathological conditions (Bouvier et al., 2015; Ma et al., 2020), and in neurodegenerative diseases (Zhuang et al., 2023). However, the non-linear model-fitting of the complex qBOLD model has been a challenge in data with a limited signal-to-noise (SNR) ratio. To overcome some of these limitations, Bayesian approaches offer a robust framework for model fitting that has proven effective in similarly complex applications, such as perfusion estimation using Arterial Spin Labeling (Chappell et al., 2009, 2010). For this study, we used a qBOLD approach that enables voxelwise estimation of tissue OEF from asymmetric spin echo (ASE) scans, providing a useful method for assessing brain regions that may be impacted by aging and cognitive impairment. Building upon the Variational Bayesian qBOLD framework proposed by Cherukara et al. (2019), we integrated prior knowledge about physiological parameters, which can be derived from both imaging and non-imaging data, to regularize the fitting process and improve parameter estimation.

In the present study, we first evaluated numerical simulations of ASE data across varying physiological conditions to optimize qBOLD model fitting for accurate OEF estimation, employing a variational Bayesian method to incorporate prior knowledge. Then, we quantified OEF maps non-invasively in older adults with and without amnestic mild cognitive impairment using ASE MRI scans combined with qBOLD modeling, allowing estimation of regional differences in brain oxygen metabolism. Finally, the study examined the relationship between OEF, age, and episodic memory, a cognitive domain particularly vulnerable in the progression of both typical aging and cognitive impairment, among a group of cognitively normal (CN) and mild cognitive impairment (MCI) individuals. This approach allowed for the assessment of the potential of OEF as a biomarker for cognitive decline and brain aging.

## 2 Theory of Two-Compartment Quantitative BOLD (qBOLD) Model

The qBOLD model assumes that the blood vessels in each voxel are uniformly and randomly distributed (Yablonskiy & Haacke, 1994). The theoretical framework describes the evolution of MR signals in biological tissue under static magnetic field inhomogeneities, corresponding to the static dephasing regime. A complete two-compartment qBOLD model was introduced to explicitly account for intravascular (blood) and extravascular (tissue) signal contributions (He & Yablonskiy, 2007). In this framework, R_2_^’^-weighted measurements can be achieved using multiple ASE acquisitions (An & Lin, 2003) and fit to the qBOLD model across echo times to measure OEF for each voxel (Cherukara et al., 2019).

The qBOLD model yields a relation between R_2_^’^, OEF and DBV (An & Lin, 2000; He & Yablonskiy, 2007):

$${R^{\prime }}_{\text{2}}\;=\mathrm{DBV}\cdot \delta \omega =\frac{\text{4}}{\text{3}}\pi \cdot \mathrm{DBV}\cdot \gamma \cdot \Delta \chi_{\text{0}}\cdot \mathrm{Hct}\cdot {\mathrm{OEF}}_{\text{0}}\cdot \mathrm{OEF}\;$$ (1)

where δω is the characteristic frequency determined by the magnetic susceptibility difference between deoxygenated blood and the surrounding tissue, as defined in Eq. (2); γ is the gyromagnetic ratio for water proton (γ = 2.68 x 10^8^ rad/s/T)*;* Δχ*_0_* is the magnetic susceptibility difference between fully oxygenated and fully deoxygenated red blood cells ($\Delta \chi_{0}\;=0.264\;$ ppm) (Spees et al., 2001); *Hct* is the blood fractional hematocrit, assumed to be 0.40 for all participants (Nicoll et al., 2012); *B_0_* is the static magnetic field strength (*B_0_* *=* 3 T); and DBV refers to deoxygenated blood volume, which is primarily contributed by venous blood.

The goal of qBOLD modeling is to disentangle the effects of OEF and DBV from the ASE signal data to produce voxelwise OEF maps. The ASE signals are R_2_^’^-weighted and exhibit non-monoexponential signal decay, with two distinct analytical expressions depending on the echo-time regime (An & Lin, 2000; Yablonskiy & Haacke, 1994). Specifically, the R_2_^’^-weighted signal behavior can be divided into two different regimes of the spin echo displacement time, τ. The boundary between the two different regimes is defined at T_C_ = 1.76 t_C_ (Cherukara et al., 2019), where t_C_ is the characteristic time. This characteristic time is the inverse of the characteristic frequency, which is given as (H. Lee et al., 2018).

$$\frac{\text{1}}{t_{c}}=\delta \omega =\frac{\text{4}}{\text{3}}\pi \cdot \gamma \cdot \Delta \chi_{\text{0}}\cdot \mathrm{Hct}\cdot {\mathrm{OEF}}_{\text{0}}\cdot \mathrm{OEF}\;$$ (2)

The signal originating from the extravascular tissue compartment can be described by two asymptotic functions of τ:

$${\text{S}}^{t}\left(\tau \right)=\{\begin{array}{c} \text{C}\cdot \text{exp}\left(-\frac{\text{3}}{\text{10}}\cdot \mathrm{DBV}\cdot {\left(\delta \omega \cdot \tau \right)}^{\text{2}}\right),\;\;\;\;\;\;\;\;\;\;\left|\tau \right|<T_{C}\\ C\cdot \text{exp}\left(\mathrm{DBV}-\tau \cdot \mathrm{DBV}\cdot \delta \omega \right),\;\;\;\;\;\;\;\;\;\left|\tau \right|>T_{C} \end{array}$$ (3)

For the regime of |τ|<T_c_, signal decay is dominated by reversible dephasing due to microscopic susceptibility variations and the tissue signal ${\text{S}}^{t}\left(\tau \right)$ depends quadratically on τ. In contrast, in the regime of |τ|>T_c_, the signal decay becomes approximately monoexponential, with a slope governed by the reversible transverse relaxation rate (R_2_^’^). Given ${R^{\prime }}_{2}=\mathrm{DBV}\cdot \delta \omega$ (Eq. 1), the temporal evolution of the tissue signal, $S^{t}$, can be reformulated in terms of R_2_^’^, thereby eliminating explicit dependence on OEF from Eq. (3):

$${\text{S}}^{t}\left(\tau \right)=\{\begin{array}{c} C\cdot \text{exp}\left(-\frac{\text{3}}{\text{10}}\frac{{\left({R^{\prime }}_{\text{2}}\tau \right)}^{\text{2}}}{\mathrm{DBV}}\right),\;\;\;\;\;\;\;\;\;\;\left|\tau \right|<T_{C}\\ C\cdot \text{exp}\left(\mathrm{DBV}\;-\;{R^{\prime }}_{\text{2}}\cdot \tau \right),\;\;\;\;\;\;\;\;\;\left|\tau \right|>T_{C} \end{array}\;\;$$ (4)

where $C={\text{S}}_{\text{0}}\cdot \text{exp}\left(-R_{\text{2}}^{t}\cdot \text{TE}\right)$ is a voxelwise baseline signal amplitude at the fixed echo time TE. Here, ${\text{S}}_{\text{0}}$ accounts for constant terms of signal intensity, TE is the spin echo time, which is constant across all acquired ASE echoes, and $R_{2}^{t}$ is the irreversible transverse relaxation rate of bulk tissue, which was assumed to be 11.5 (s^-1^) (He & Yablonskiy, 2007).

For the intravascular signals, assuming a random distribution of vessel orientations within each voxel, the corresponding frequency shifts are also treated as uniformly distributed. Under this assumption, the normalized blood signal, $S^{b}$, can be expressed as (Sukstanskii & Yablonskiy, 2001; Yablonskiy et al., 2013):

$$\begin{array}{l} {\text{S}}^{\text{b}}\left(\tau \right)=\text{exp}\left(-R_{\text{2}}^{\text{b}}\cdot \text{TE}+\text{i}\frac{\delta \omega \cdot \tau }{\text{2}}\right)\\ \;\;\;\;\;\;\;\cdot \frac{C\left({\left(\frac{\text{3}\delta \omega \left|\tau \right|}{\pi }\right)}^{\text{2}}\right)-\text{iS}\left({\left(\frac{\text{3}\delta \omega \left|\tau \right|}{\pi }\right)}^{\text{2}}\right)}{\left({\left(\frac{\text{3}\delta \omega \left|\tau \right|}{\pi }\right)}^{\text{2}}\right)}\; \end{array}$$ (5)

where *C(x)* and *S(x)* are Fresnel integral functions, and δω is the characteristic frequency shift as defined in Eq. (1). The transverse relaxation rate of blood within the intravascular compartment, $R_{2}^{b},\;$ is influenced by the blood hematocrit (*Hct*), the proportion of red blood cells in the blood volume, and by OEF (Simon et al., 2016):

$$R_{\text{2}}^{\text{b}}=\left(\text{16.4}\mathrm{Hct}+\text{4.5}\right)+\left(\text{165.2}\mathrm{Hct}+\text{55.7}\right)\;\cdot \;\text{OE}{\text{F}}^{\text{2}}$$ (6)

The total signal calculated from a voxel in this two-compartment tissue model is the sum of the signal from each compartment:

$${\text{S}}^{\text{Total}}\left(\tau \right)={\text{S}}_{\text{0}}\left(\mathrm{DBV}\cdot {\text{S}}^{\text{b}}\left(\tau \right)+\left(\text{1}-\mathrm{DBV}\right)\cdot {\text{S}}^{t}\left(\tau \right)\right)\;\;$$ (7)

This equation can be used as a forward model in a Bayesian framework to infer DBV and R_2_^’^ parameters, enabling faster and more reliable model fitting by incorporating prior information to produce more reasonable estimates in the presence of noise (as described in Supplementary Methods). The Variational Bayes (VB) method provides an efficient way to optimize and select a prior distribution for DBV and R_2_^’^, including those for mean μ_0_ and standard deviations $\sigma_{0}$ (Chappell et al., 2009). OEF is later calculated from the estimated DBV and R_2_^’^ parameters by rearranging parameters in Eq. (1) to achieve Eq. (8). A full schematic of the qBOLD pipeline is shown in Figure 1:

**Fig. 1.**
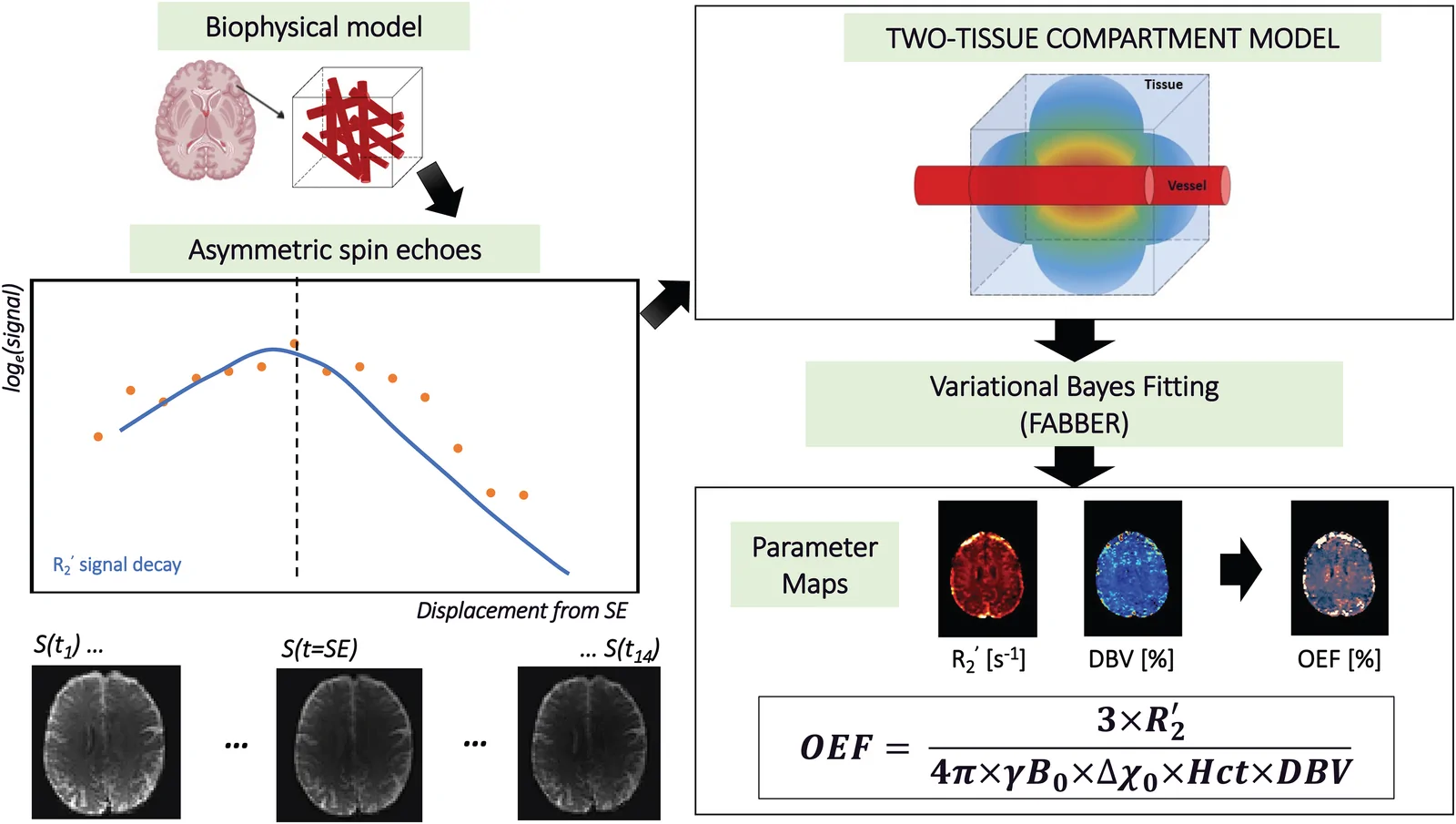
Schematic of qBOLD describing the pipeline of quantifying OEF maps using Asymmetric Spin Echo (ASE) sequence using a two-tissue compartment model in a Bayesian framework. Abbreviations: SE, spin echo; FABBER: FAST ASL and BOLD Bayesian Estimation Routine, R_2_^’^: Transverse Relaxation Rate; DBV: Deoxygenated Blood Volume; OEF: Oxygen Extraction Fraction.

$$\mathrm{OEF}=\frac{\text{3}}{\text{4}}\cdot \frac{{R^{\prime }}_{\text{2}}}{\mathrm{DBV}\cdot \gamma \cdot \pi \cdot \Delta \chi_{\text{0}}\cdot \mathrm{Hct}\cdot {\mathrm{OEF}}_{\text{0}}}$$ (8)

## 3 Materials and Methods

### 3.1 Optimization for prior standard deviations

Following the approach of Cherukara et al. (2019), simulated qBOLD data were used to estimate an appropriate prior using a Bayesian model-based approach, and to assess whether incorporating this prior improves OEF estimates compared to using no prior. Since Bayesian model fitting relies on prior distributions to regularize parameter estimations, the standard deviations of these priors were optimized to constrain the parameter estimates while maintaining fidelity to the measured data, thereby improving the accuracy and stability of qBOLD parameter estimates. Two-compartment ASE qBOLD signals were simulated (An & Lin, 2003; Berman et al., 2018; He & Yablonskiy, 2007) in MATLAB (MathWorks, Natick, MA, 2021) for a range of underlying parameters covering different physiological regimes. Simulations were generated for 900 artificial ASE signals, including 30 OEF values from 20% to 70% and 30 DBV values from 0.3% to 15%. For later analysis, the full DBV range was divided into three physiological ranges: low DBV (0.3–2.5%), medium DBV (2.5–7.5%), and high DBV (7.5–15%). Gaussian noise was added to achieve a signal-to-noise ratio (SNR) of 70 that matched the corresponding images in our *in vivo* dataset.

The full set of simulated signals was analyzed using R_2_^’^-DBV-based qBOLD models (Eq. 7) with the VB scheme. Estimation of (R_2_^’^, DBV) produces more distinct parameters compared to direct estimation of (OEF, R_2_^’^) in qBOLD models and can be more readily approximated by a multivariate normal distribution (Cherukara et al., 2019). This analysis was conducted using the FAST ASL and BOLD Bayesian Estimation Routine (FABBER) (Chappell et al., 2009; Groves et al., 2009; Woolrich & Behrens, 2006) using the two-tissue compartment qBOLD models with output parameters of DBV and R_2_^’^ (He & Yablonskiy, 2007). Prior means (µ_0_) were taken from the literature for healthy subjects (µ_0_ (DBV) = 3.6% and µ_0_ (R_2_^’^) = 2.6 s^-1^) (Stone & Blockley, 2017). We evaluated different standard deviations (σ_0_) of the prior distribution. VB inference was performed using a two-compartment (2C) model with a range of 10^-1/2^ to 10^5/2^ for each parameter (DBV and R_2_^’^) with fixed µ_0_. The estimated OEF was then calculated using Eq. (8) and constrained between 0% and 100% to restrict OEF values to a reasonable physiological range. In addition, DBV was restricted to values greater than 0%, and R_2_^’^ was constrained to be greater than 0 s^-1^. The absolute error for each parameter (compared to the simulated ground truth) was then averaged over all simulated pairs of OEF and DBV values to identify the optimal σ_0_ settings that gave the lowest error across all physiological ranges.

### 3.2 Participants

70 older participants (74.7 ± 6.9 years old, 24 males, and 46 females) were recruited for MRI, including 12 patients with amnestic MCI and 58 CN individuals with detailed characteristics shown in Table 1. The study protocol was approved by the Institutional Review Board at the University of California, Davis. All participants provided written informed consent before participating in this study. In addition, participants who could not receive an MRI due to the inability to lie motionless in the scanner, pacemakers, aneurysm clips, neurostimulators, artificial heart valves, metal objects in the eyes, ear implants, or claustrophobia were excluded.

**Table 1.** Participant characteristics.

	Whole group	CN	MCI	Uncorrected *P*-value
N	70	58	12	-
Age (years)	74.7 ± 6.9	74.5 ± 7.1	75.6 ± 5.8	0.50
Sex (number of females)	46 (65.7%)	38 (65.5%)	8 (66.7%)	0.94
Education (years)	14.2 ± 4.6	14.6 ± 3.8	12.2 ± 7.4	0.51
Episodic memory (z-score)	-0.17 ± 0.88	0.41 ± 0.74	-0.92 ± 0.63	<0.0001*
WMH volume (cm^3^)	3.17 ± 4.49	2.93 ± 4.27	4.33 ± 5.49	0.18
Left hippocampal volume (cm^3^)	3.05 ± 0.36	3.09 ± 0.33	2.83 ± 0.39	0.02*
Right hippocampal volume (cm^3^)	2.91 ± 0.44	2.94 ± 0.42	2.79 ± 0.53	0.35
Vascular risk score	0.16 ± 0.12	0.15 ± 0.12	0.19 ± 0.11	0.30

Note: Unless otherwise specified, data are in the form of mean ± standard deviation. Data in parentheses are percentages. Demographic characteristics were compared using Chi-squared (for sex) and Wilcoxon rank sum (for continuous variables). For dataset with cognitive performance, the scan date and the date for cognitive tests were within 6 months.

* Indicates significant *P*-value, *P* < 0.05.

CN: Cognitively normal; MCI: mild cognitive impairment; WMH: White matter hyperintensities.

The diagnosis of mild cognitive impairment was made based on the current consensus conference using standard diagnosis criteria (McKhann et al., 2011; Winblad et al., 2004). All subjects were recruited from the Alzheimer’s Disease Research Center at the University of California, Davis (ADRC). Information about vascular risk score (VRS) and white matter hyperintensity (WMH) volume from FLAIR (fluid attenuated inversion recovery) MRI scans was included as additional participant characteristics in Table 1. Methods to measure vascular risk (DeCarli et al., 2019) and white matter hyperintensity volume (DeCarli et al., 2005; Maillard et al., 2022, 2024) are included in Supplementary Methods.

### 3.3 Cognitive assessment

The primary cognitive outcome of this study was a composite measure of episodic memory derived from the Spanish English Neuropsychological Assessment Scale (SENAS) within 6 months of the MRI scan in a total of 67 subjects with 55 CN and 12 MCI subjects (3 participants in the CN group were excluded due to that criterion). SENAS is a well-developed cognitive test designed to assess cognitive aging and enable valid comparisons across race/ethnic groups (Mungas et al., 2004, 2005). The episodic memory composite score was based on performance in a multi-trial word-list-learning test (Mungas et al., 2004). These measures were administered during all evaluations, with the language of administration determined by a participant-specific algorithm considering language preferences across various contexts. Cognitive scores were created for episodic memory by computing a z-score for each test score. Details about SENAS development and psychometric characteristics are available in previous work (Mungas et al., 2004).

### 3.4 MRI data acquisition

All 70 participants were scanned using a 3T Tim Trio system (Siemens Healthcare, Erlangen, Germany) with a 32-channel head-coil. Foam padding was wrapped around the participant’s head to minimize motion during the scan. All experimental studies were approved by the Institutional Review Board of the University of California, Davis.

Data were acquired in all participants using the Gradient Echo Slice Excitation Profile Imaging Asymmetric Spin Echo (GASE) sequence with an echo planar imaging (EPI) readout having specialized z-shim gradients to mitigate within-slice inhomogeneities (Blockley & Stone, 2016). The sequence parameters included repetition time (TR) = 2.8 s; spin echo time (TE) = 66 ms; matrix size = 96 x 96 with 80 slices; spatial resolution = 2.3 x 2.3.x1.3 mm^3^; and field of view (FOV) = 224 mm. The acquisition included 14 R_2_^’^-weighted images with spin echo displacement times (τ) from 0 ms to 52 ms in the step of 4 ms, including the spin echo scan at τ =0. To provide whole-brain coverage, the acquisition was performed using twenty 3D slabs. Each slab contains four slices at 1.25-mm thickness (80 slices in total) to correct for macroscopic field gradient and mitigate through-slice signal loss. The total acquisition time of the main GASE sequence was 3 min 50 s to cover the whole brain. All primary ASE images were obtained in a phase-encoding direction (i.e., A>>P direction). A separate spin-echo image (τ=0) with the same image resolution and coverage was also obtained with the reversed phase-encoding direction (i.e., P>>A direction) for distortion correction.

T_1_-weighted anatomic scans were acquired using a multi-echo magnetization-prepared rapid gradient echo (MPRAGE) with the following acquisition parameters: TR = 2.3 s; TE = 2.98 ms; inversion time (TI) = 900 ms; spatial resolution = 1.0 x 1.0 x 1.0 mm^3^; in-plane matrix = 256 x 256; 176 slices with thickness = 1.0 mm; flip angle = 9.0°; and acquisition time of 5 min and 12 s. Fluid-attenuated inversion recovery (FLAIR) scans used a 3D acquisition with the following acquisition parameters: sagittal FOV = 256 mm; TR = 4.8 s; TE = 446 ms; TI = 1.65 s; spatial resolution = 1.0 x 1.0 x 1.2 mm^3^; and acquisition time of 4 min and 21 s.

### 3.5 Image analysis

Structural MR images were processed to be skull-stripped by using a Convolutional Neural Network (CNN) deep-learning method (Fletcher et al., 2021). All structural MR brain images were then non-linearly registered to a minimal deformation template (MDT) (Kochunov et al., 2002). Brain regions of interest (ROI) segmentations were performed in the MDT template based on the Desikan-Killiany-Tourville (DKT) atlas (Klein & Tourville, 2012), resulting in a total of 62 gray matter regions, with the left and right hemispheres analyzed separately. We additionally included the hippocampus since this region is known to be associated with episodic memory (Ranganath, 2010) resulting in a total of 64 cortical regions for the final analysis. Finally, native ROIs were computed by applying the inverse transformation of MDT brain ROIs into individual T_1-_structural space with b-spline registration parameters.

GASE data for qBOLD modeling were preprocessed in FSL (Jenkinson et al., 2012). To obtain EPI distortion-corrected images, we utilized FSL’s *TOPUP* tool to estimate the susceptibility-induced field from two spin echo images in both phase-encode directions (Andersson et al., 2003; Smith et al., 2004). Then, we used *APPLYTOPUP* to correct distortions for the rest of the R_2_^’^-weighted images. After distortion correction, GASE data were motion-corrected to the spin echo volume using *MCFLIRT* (Jenkinson et al., 2002), and smoothed with a Gaussian kernel of σ = 1.5 mm (Jenkinson et al., 2012). Finally, the spin-echo image was skull-stripped using the *Brain Extraction Tool (BET)* (Smith, 2002) to create a binary mask of the brain, which was applied to the rest of the R_2_^’^-weighted volumes.

For the *in vivo* study, GASE data were analyzed using the R_2_^’^-DBV two-compartment (2C) model in VB using FABBER with optimal standard deviation priors from the simulation study to generate parameter estimates (including DBV, R_2_^’^, and OEF maps) and compare with the 2C model without prior. To further assess regional OEF measures at the group level, the resulting quantitative OEF maps were linearly co-registered to subject-specific T_1_-structural space with FMRIB’s Linear Image Registration Tool (FLIRT) (Greve & Fischl, 2009; Jenkinson & Smith, 2001; Jenkinson et al., 2002). To account for potential confounding from partial volume effect (PVE) in regions susceptible to tissue mixing, gray matter probability maps were generated via tissue segmentation using the statistical parametric mapping software (SPM 12, http://www.fil.ion.ucl.ac.uk/spm/software/spm12/) (Penny et al., 2011). All subsequent analyses were restricted to voxels within a gray matter mask, thresholded at a probability value of 0.2 (i.e., voxels with > 20% likelihood of being gray matter), to minimize contamination from adjacent white matter and cerebrospinal fluid.

### 3.6 Statistical analysis

#### 3.6.1 Regional analysis

All statistical analyses were performed using MATLAB 2021a (MathWorks, Natick, MA, 2021).

For *in vivo* OEF maps, voxels with non-physiological extreme values, defined as those lower than 0% and greater than 100%, were excluded from the analysis, consistent with previous studies (Cherukara et al., 2019; Stone & Blockley, 2017). For regional analysis, the mean for each ROI (including both left and right hemispheres) was calculated for each participant. To examine group differences in OEF in each region, we performed a linear regression analysis with OEF as a dependent variable and diagnostic group (CN versus MCI) as the independent variable. Regional brain volume was included as a covariate to control for anatomical differences between subjects. False Discovery Rate (FDR) correction (Groppe et al., 2011) was applied to adjust for multiple comparisons across a total of 64 regions. A two-tailed P<0.05 was considered statistically significant.

#### 3.6.2 Relationship of OEF with age and cognitive performance

To evaluate the relationship of OEF with age in each ROI (Model 1), multiple linear regression analysis was conducted using OEF as the dependent variable and age, diagnosis, and sex as the independent variables. Sex was treated as a categorical variable (Sex: 1 = Male, 2 = Female). Diagnosis was coded as -1 and 1 for CN and MCI participants, respectively, to measure an overall age effect across diagnostic groups. If a significant interaction (Age×Diagnosis) effect was observed, post-hoc multiple linear models were performed with diagnosis as a categorical variable to investigate the relationship between OEF and age for each diagnostic group.

For the association between imaging and cognition (Model 2), we also used a multiple linear regression model, in which the cognitive test scores (i.e. episodic memory) were dependent variables, and OEF was the independent variable. Sex, education, and age were the covariates. The interaction between diagnosis and OEF was also tested (OEF×Diagnosis ). If a significant interaction effect was observed, we then also conducted analyses investigating the effect of OEF and cognition for each diagnostic group.

In all regression analyses between OEF with age and cognition, regional volume was included as an additional covariate in the model, with each ROI’s volume normalized to the total brain volume for that subject to account for inter-subject differences. All OEF measures were corrected for PVE prior to statistical analysis. Finally, FDR correction was conducted for multiple regional comparisons.

For brain region visualization, we utilized the toolbox ITK-SNAP (Madan, 2015) to display anatomical location of the selected regions of interest.

## 4 Results

### 4.1 Standard deviation prior optimization

Simulated ASE signals were fit using the 2C qBOLD model in the VB framework to evaluate error in parameter estimations. A range of standard deviations for the priors on R_2_^’^ and DBV (from 10^-1/2^ to 10^5/2^) was tested to determine optimal prior information. The results are shown in the heatmap in Supplementary Figure S1, which suggests that σ_0_ (R_2_^’^) = 10^3/2^ and σ_0_ (DBV) = 10^1/2^ are an optimal combination that produces the smallest error across the various physiological ranges. Across the DBV regimes, the effect of prior on OEF and DBV errors converged quickly at low prior σ_0_ (R_2_^’^) and remained consistent after σ_0_ (R_2_^’^) = 10 for all σ_0_ (DBV). Therefore, these findings led us to choose the prior combination of σ_0_ (R_2_^’^) = 10^3/2^ and σ_0_ (DBV) = 10^1/2^ for later *in vivo* analyses.

### 4.2 *In vivo* human studies

Participant characteristics are presented in Table 1. Using Chi-squared and Wilcoxon analysis, we found no significant differences in age (P=0.50 ), sex (P=0.94 ), education (P=0.51 ), WMH volume (P=0.18 ), right hippocampal volume (P = 0.35), and vascular risk score (P=0.30 ) between the two diagnostic groups. However, there were significant differences in episodic memory (P<0.001 ) and left hippocampal volume (P=0.02) between the groups.

#### 4.2.1 In vivo quantitative parameter maps

The SNR for each ROI was calculated by dividing the mean of GASE voxel intensity after preprocessing within the region by the standard deviation of the noise outside the head. Across all regions, all subjects, and τ  values, the average SNR was 60.9 ± 13.4. The SNR for each region was reported in Supplementary Table S1.

To visualize the effects of the optimized prior on the *in vivo* data, all three parameter maps were shown in a representative participant (cognitively normal male, 80 years old). Figure 2 demonstrates the improvement of parameter maps (DBV, R_2_^’^, and OEF) estimated with optimal prior standard deviations in this participant. Use of the prior visually reduced the presence of extreme DBV and OEF values (bright voxels) in the cortical regions (i.e. those with OEF more than 100%, with DBV more than 50% and with R_2_^’^ more than 20 s^-1^). Across all subjects and all regions, the average percentage of voxels with unphysiological OEF estimates was 7.3 ± 8.1% using the 2C model with optimal prior standard deviations, compared to 17.1 ± 9.7% without prior information. Similar improvements were observed for DBV and R_2_^’^ estimations. Without prior information, 17.4 ± 7.7% (DBV) and 6.0 ± 6.2% (R_2_^’^) of voxels were removed, while with an optimal prior, only 4.3 ± 4.8% (DBV) and 2.2 ± 5.0% (R_2_^’^) were excluded. Details on the percent of voxels removed due to poor qBOLD model fits in each region are reported in Supplementary Table S1.

**Fig. 2.**
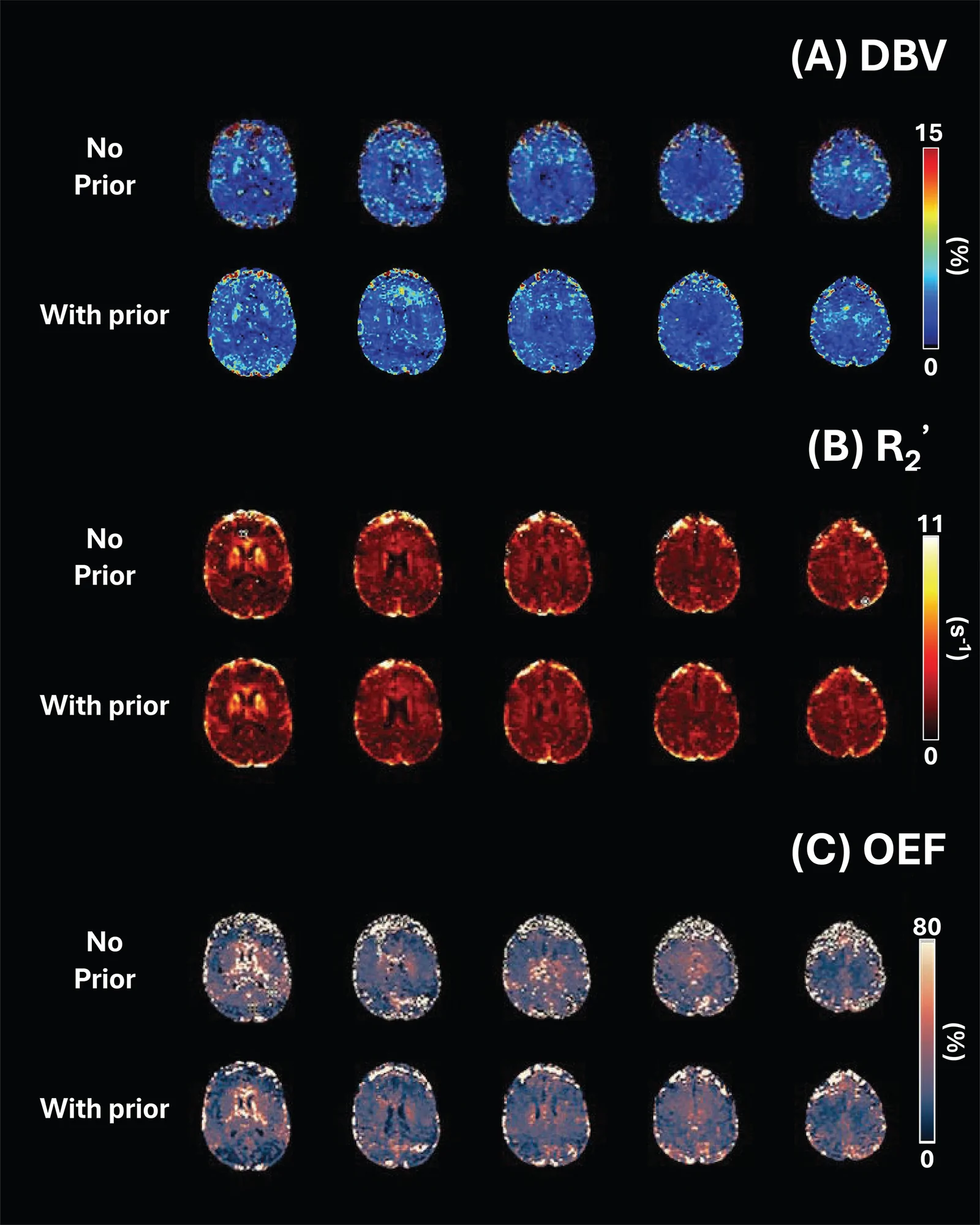
Parameter maps from a representative cognitively normal male participant (80 years old), including (A) deoxygenated blood volume (DBV in %), (B) transverse relaxation rate (R_2_^’^ in s^-1^), and (C) oxygen extraction fraction (OEF in %), comparing two-tissue (2C) compartment models with (bottom row for each panel) and without (top row for each panel) prior optimization.

#### 4.2.2 Difference of OEF between diagnostic groups

The group-average OEF values (separately from CN and MCI groups) quantified from qBOLD modeling across all participants in different regions are shown in Figure 3, using VB framework with optimal priors. Compared to the CN group, increased average OEF was observed in the MCI group across diffuse cortical regions in the brain. Mean OEF values for all subjects across all regions for each group are presented in Supplementary Table S2. Using a linear regression model, we observed the largest OEF elevations in the MCI group in left (L) and right (R) cuneus (P=0.032 , and P=0.0.031 , respectively), L inferior parietal (P=0.024 ), L lateral occipital (P=0.041 ), L and R pericalcarine (P=0.030 and P=0.010 , respectively), L precuneus (P=0.029 ), R lingual (P=0.036) , and R superior frontal (P=0.033 ). However, no region showed a statistically significant difference between the two groups after FDR correction. Of note, we also observed a trend of decreased OEF in the MCI group compared to the CN group in some regions, especially in entorhinal (L: ΔOEF=−3.92%, P=0.160 ; R: ΔOEF=−2.20%, P= 0.414) , parahippocampal (L: ΔOEF=−2.71%,  P=0.103 ; R: ΔOEF=−0.81%, P=0.589 ), and hippocampus (L: ΔOEF=−2.82%, P=0.162 ; R: ΔOEF=−1.50%,  P=0.404 ), although none were significantly different.

**Fig. 3.**
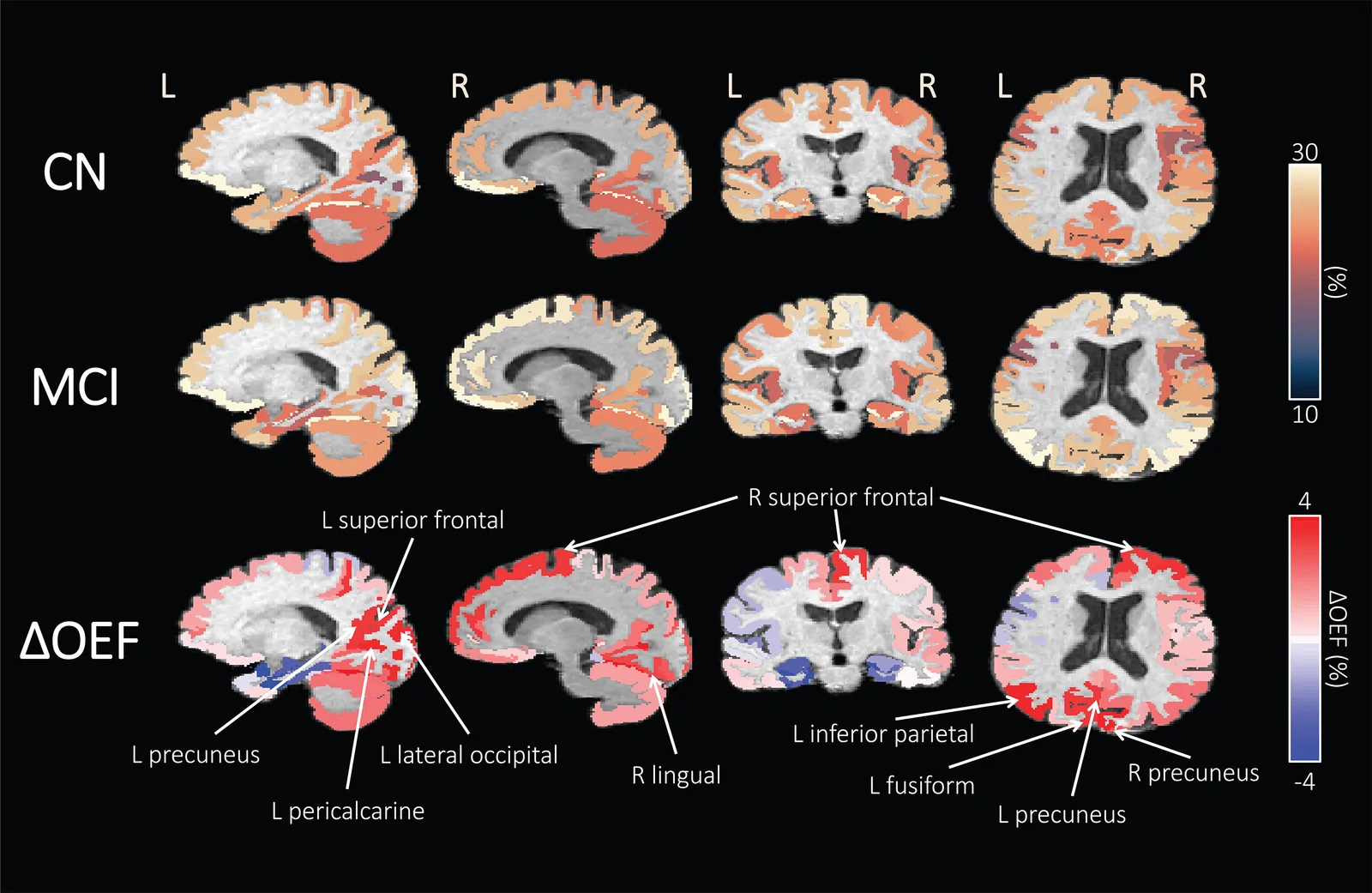
Oxygen Extraction Fraction (OEF in %) maps in group level in (top row) cognitively normal (CN) (N = 58) and (middle row) Mild-Cognitive-Impairment (MCI) (N = 12) subjects, and (bottom row) the absolute difference of OEF between two diagnostic groups (ΔOEF in %). All regions include both left (L) and right (R) hemispheres. Regions that show significant differences between the two groups before FDR correction are labeled (with white arrows) in the figure.

#### 4.2.3 Age showed correlations with OEF in the mild cognitively impaired group

Given data indicating age-related differences in OEF, we investigated the relationship between OEF and age in each ROI using multiple linear regression analysis. A significant Age×Diagnosis interaction was observed in the left inferior parietal region (β=0.55±0.13, T=4.43,  P<0.0001 ), which survived FDR correction for multiple regional comparisons (adjustedP=0.002) . A similar interaction effect was observed in the right inferior parietal region (β=0.39±0.14, T=2.84, P=0.006 ); however, this effect did not survive FDR correction. Therefore, we conducted further analyses for each diagnostic group. We observed that in the MCI group, older age was associated with increased OEF in both left inferior parietal (β=0.88±0.23, T=3.75, P=0.0004 ) and right inferior parietal areas (β=0.71±0.25, T=2.81, P=0.007 ) (Fig. 4, Table 2). However, the opposite trend was observed in the CN group in the left inferior parietal region (β=−0.23±0.09, T=−2.70, P=0.00 9). There was no significant association with age in the right inferior parietal region (β=−0.06±0.09, T=−0.64, P=0.524 ). After FDR correction for multiple regional comparisons, there remained a significant increase in OEF with older age in the MCI group in the left inferior parietal (adjusted P=0.024 ) region. There were no significant associations between OEF and age in other brain regions for either the CN or MCI group after FDR correction. Results for other regions are shown in Supplementary Table S3.

**Fig. 4.**
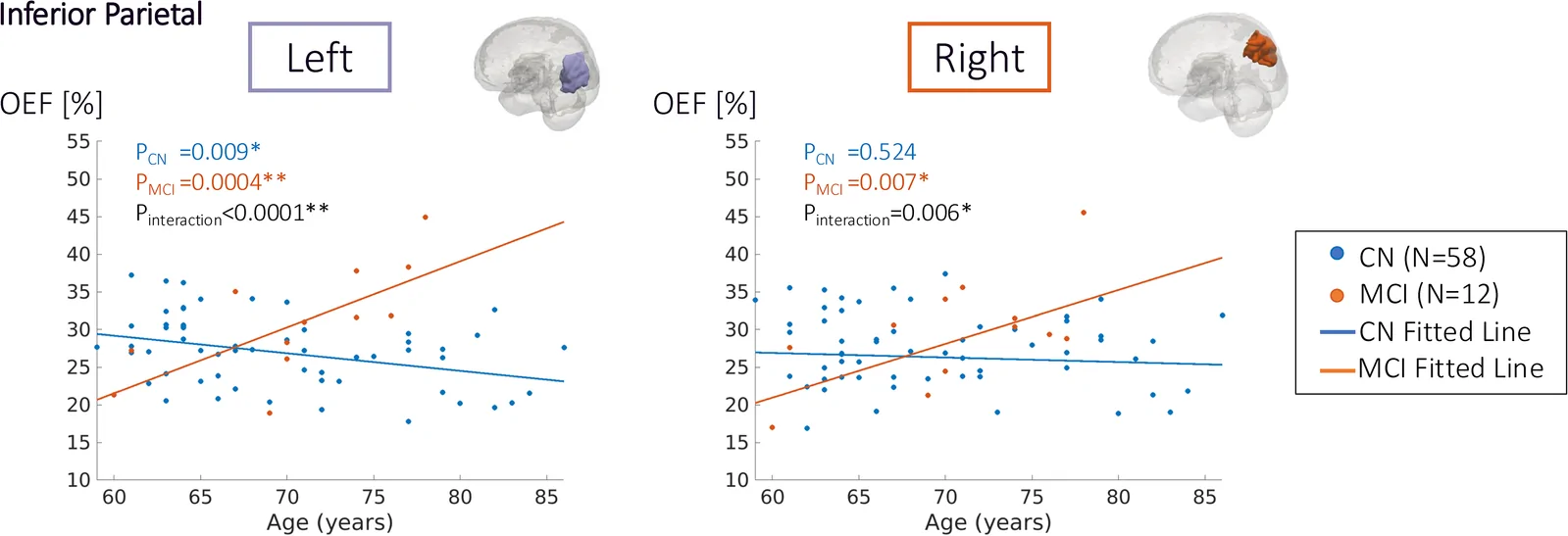
The correlation between oxygen extraction fraction (OEF in %) and age (in years) in cognitively normal (CN, blue) and Mild Cognitive Impaired (MCI, orange) groups in left and right inferior parietal regions. OEF values were corrected for partial volume effects. Multiple linear regression model included sex, diagnosis, and regional volume as covariates (Section 3.6.2). (*indicates significant uncorrected *P*-value, P < 0.05, **indicates significant *P*-value after FDR correction)

**Table 2.** Multi-linear regression models between OEF, age, and diagnosis in the whole group (N = 70) in left and right inferior parietal regions.

	Left inferior parietal			Right inferior parietal		
Effect	β ± SE	T	P-value	β ± SE	T	P-value
Age	0.32 ± 0.12	2.60	0.012	0.33 ± 0.13	2.43	0.018
can	-0.23 ± 0.09	-2.70	0.009	-0.06 ± 0.09	-0.64	0.524
MCI^a^	0.88 ± 0.23	3.75	0.0004	0.71 ± 0.25	2.81	0.007
Region volume	-2.75 ± 10.12	-0.27	0.786	-6.93 ± 11.05	-0.63	0.533
Sex	0.36 ± 1.14	0.31	0.756	1.81 ± 1.25	1.45	0.151
Diagnosis	-39.87 ± 9.47	-4.21	<0.0001	-28.17 ± 10.33	-2.73	0.008
Age x diagnosis	0.55 ± 0.13	4.43	<0.0001	0.39 ± 0.14	2.84	0.006

In this model, OEF was the dependent variable. The independent variables are listed above.

a The analysis of the multi-linear regression model between OEF and age in each group, with diagnosis as a categorical variable.

CN: cognitively normal; MCI: mild cognitive impairment; β: standardized beta coefficient; SE: standardized error; OEF: oxygen extraction fraction.

#### 4.2.4 Relationship of OEF with episodic memory

When examining the association between OEF and episodic memory cognitive scores, we found significant interaction effects (*OEF×Diagnosis*) in both left (L) and right (R) parahippocampal regions (L: β=0.06±0.02, T= 2.82, P=0.007 ; R: β=0.09±0.02, T=3.77, P=0.0004 ) (Table 3) before FDR correction for multiple comparisons. The interaction effect between the two diagnostic groups and OEF in the right parahippocampal region persisted after FDR correction for multiple comparisons (adjusted P=0.024 ). Additionally, in Supplementary Table S4, we also observed significant (*OEF×Diagnosis*) interactions—prior to FDR correction—in the association between OEF and episodic memory scores, particularly in regions within the frontal, temporal, and parietal lobes.

**Table 3.** Multi-linear regression model between OEF and episodic memory in the whole group (N = 67) in left and right parahippocampal regions.

	Left parahippocampal			Right parahippocampal		
Effect	β ± SE	T	P	β ± SE	T	P
OEF	0.01 ± 0.02	0.64	0.525	0.06 ± 0.02	2.60	0.012
Can	-0.04 ± 0.02	-2.89	0.005	-0.03 ± 0.02	-1.66	0.103
MCI^a^	0.07 ± 0.04	1.88	0.065	0.15 ± 0.04	3.42	0.001
Age	-0.002 ± 0.010	-0.21	0.837	-0.005 ± 0.010	-0.45	0.652
Education	0.04 ± 0.02	2.77	0.007	0.07 ± 0.02	4.14	0.0001
Region volume	-1.93 ± 10.51	-0.18	0.797	-5.11 ± 10.51	-0.49	0.629
Sex	0.70 ± 0.15	4.64	<0.0001	0.75 ± 0.15	5.15	<0.0001
Diagnosis	-1.91 ± 0.46	-4.20	<0.0001	-2.62 ± 0.54	-4.87	<0.0001
OEF x diagnosis	0.06 ± 0.02	2.82	0.007	0.09 ± 0.02	3.77	0.0004

In this model, OEF was the independent variable, while age, education, sex, regional volume and diagnosis were covariates. OEF values were corrected for partial volume effects.

a The analysis of the multi-linear regression model between episodic memory and OEF in each group, with diagnosis as a categorical variable.

CN: cognitively normal; MCI: mild cognitive impairment; β: standardized beta coefficient; SE: standardized error; OEF: oxygen extraction fraction.

We then further investigated the relationship between episodic memory scores and OEF in different diagnosis groups using the same model with diagnosis as a categorical variable. OEF was inversely correlated with episodic memory for CN in the left parahippocampal region (β=−0.04±0.02, T=−2.89, P=0.005 ). However, there was an opposite trend for MCI in the right parahippocampal region (β=0.15±0.04,T=3.42, P=0.001 ) (Fig. 5, Table 3). There were no significant associations between OEF and episodic memory in other brain regions in either CN or MCI group after FDR correction. Results for other regions are provided in Supplementary Table S4.

**Fig. 5.**
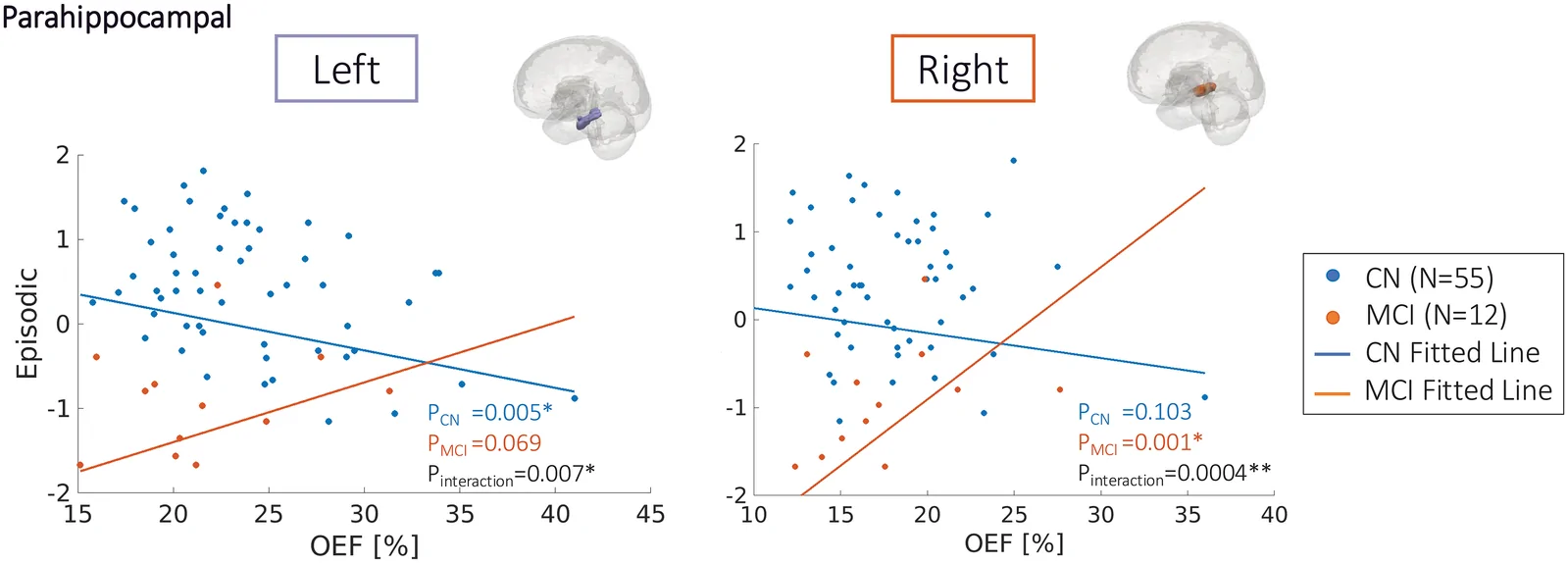
The correlation between oxygen extraction fraction (OEF in %) and episodic memory in cognitively normal (CN, blue) and mild cognitively impaired (MCI, orange) groups in left and right parahippocampal regions. OEF values were corrected for partial volume effects. Multiple linear regression model included age, sex, education, and regional volume as covariates (Section 3.6.2). (*indicates significant uncorrected *P*-value, **indicates significant *P*-value after FDR correction, P<0.05).

## 5 Discussion

This preliminary study investigated regional brain OEF, quantitatively measured from a novel non-invasive quantitative BOLD (qBOLD) MRI method, in mild cognitively impaired patients and cognitively normal individuals, and its associations with age and cognition. We first used numerical simulations to optimize the prior standard deviations and improve the fitting of the qBOLD model within the Bayesian framework. Using the optimized model for our *in vivo* SNR of 70, we found that MCI patients showed trends of higher OEF compared to the cognitively normal subjects in many cortical regions, consistent with vascular contributions to cognitive impairment. We also found that age was positively associated with OEF in many regions in mild cognitive impaired patients, significantly in the inferior parietal region. Additionally, the relationship between OEF and episodic memory within the parahippocampal regions differed significantly between diagnostic groups.

### 5.1 Regional OEF comparisons between cognitively normal and mild cognitively impaired groups

We found that MCI subjects had higher OEF than cognitively normal subjects in many cortical regions, especially in left inferior parietal (ΔOEF=3.74%, P=0.024 ), and left precuneus (ΔOEF=3.36%, P=0.029 ). This finding aligns with prior PET and MRI studies that have reported elevated OEF in parietal and temporal regions in cognitively impaired patients (Chiang et al., 2022; Frackowiak et al., 1981; Nagata et al., 2002; Tohgi et al., 1998). Previous MRI studies have explored the relationship between OEF and CBF, suggesting that capillary dysfunction may lead to reduced CBF, triggering a compensatory increase in OEF to maintain oxygen delivery to brain tissue (Chiarelli et al., 2022; Cho et al., 2020; Le et al., 2023; Nielsen et al., 2020; Peng et al., 2014). Similarly, FDG-PET studies have shown a compensatory rise in brain metabolism among individuals with MCI (Ashraf et al., 2015). Although this study did not assess CBF directly, previous research has linked hypoperfusion to MCI (Dai et al., 2009; de Eulate et al., 2017; Liu et al., 2014), which may have contributed to a compensatory increase in OEF in our MCI group.

White matter hyperintensities and vascular risk factors might also contribute to elevated OEF in individuals with MCI. WMHs, which are often observed in neuroimaging as areas of increased signal intensity on FLAIR MRI scans, are common in old age and associated with cognitive impairment (Barker et al., 2014; Bilello et al., 2015; Dadar et al., 2022; van der Flier & Scheltens, 2005; Yoshita et al., 2006) and indicative of small vessel disease and microvascular injury (Barker et al., 2014). Higher WMHs are associated with disrupted blood flow to the brain (Kang et al., 2022), which may also lead to compensatory OEF increases. Similarly, VRS incorporates factors such as hypertension, diabetes, hyperlipidemia, and smoking, all of which contribute to the likelihood of cerebrovascular damage. The compensatory OEF increase is consistent with the previous study that also reported an increase in OEF in superficial lobar areas of patients with cerebral small vessel disease (R. Zhang et al., 2025). In this study, there were no significant correlations between OEF in any brain region with WMH burden or with VRS in our cohort due to a small sample size. Nonetheless, we observed a slight elevation in WMH volume and VRS in MCI (4.33 ± 5.49 cm^3^ and 0.19 ± 0.11, respectively) compared to CN (2.93 ± 4.27 cm^3^ and 0.15 ± 0.12, respectively) (Table 1), which could indicate cerebrovascular disease contributing to cognitive decline in our MCI group. Future work will investigate the effect of different VRS and WMH levels on OEF in a larger cohort.

While most brain regions in the MCI group showed increased OEF, certain areas exhibited a reduction, as detailed in Supplementary Table S2. Notably, key regions associated with cognitive decline, such as the parahippocampal and entorhinal cortices, demonstrated lower OEF. An early PET study also observed reduced OEF in the medial temporal lobe (MTL) in patients with cognitive impairment (Ishii et al., 1996). Additionally, MRI techniques have been proposed to measure OEF in specific veins of the MTL, with findings indicating that OEF in this region is 9.4% lower than global OEF (Jiang et al., 2023). These observations may reflect regionally specific OEF reductions associated with reduced neuronal activity in the MTL, a region particularly vulnerable to early neurodegeneration and cognitive decline.

### 5.2 Regional associations of OEF with age in the mild cognitively impaired group

We found that older age showed associations with increased OEF in the left inferior parietal region in the MCI group after multiple comparisons correction. This region has been reported to be affected by both aging and cognitive impairment (Bakkour et al., 2013). Additionally, cerebral blood flow is known to decrease with age (Chen et al., 2011; Pantano et al., 1984; C. Wu et al., 2016) and with progression from CN to MCI (de Eulate et al., 2017; Mokhber et al., 2021; van Dinther et al., 2024; H. Zhang et al., 2021). Thus, in the MCI group, OEF may increase as a vascular compensatory mechanism with age to maintain cerebral metabolism in the presence of lower blood flow (Fan, An, et al., 2020).

Although the effects did not pass multiple comparisons correction, it is notable that different trends with age were observed in cognitively normal versus MCI groups across most brain regions (Supplementary Fig. S3). In healthy aging without cognitive impairment, OEF as a measure of brain metabolic function declines with age, potentially due to the accumulation of Aβ, neuronal loss (Bishop et al., 2010; Edison et al., 2007; Mormino & Papp, 2018), or other age-related factors. Our result is consistent with early studies that used PET (Eustache et al., 1995; Yamaguchi et al., 1986) and other MRI (Chiang et al., 2022) to report decreased OEF with increasing age in healthy subjects. Although other studies also reported opposite trends of increasing OEF with age (Jaroszynski et al., 2026; Jiang et al., 2023; Z. Lin et al., 2023; Lu et al., 2011), we note that the correlation between OEF and age may vary by brain regions and different MRI techniques. In addition, differences in study populations may contribute to these inconsistencies. Our cohort consisted primarily of older adults with vascular risk factors, which may influence age-related OEF patterns compared with prior studies that included younger, healthier participants or broader age ranges. Future work could investigate OEF differences across groups stratified by vascular risk and extend longitudinal observations (Z. Lin et al., 2023) beyond global OEF to regional OEF in normal aging and vascular risk.

### 5.3 Association of episodic memory with OEF in parahippocampal regions

We assessed the relationship between episodic memory and OEF across all ROIs in DKT atlas. We found significant differences between diagnostic groups in many frontal, parietal, and temporal brain regions (Supplementary Table S4) before FDR correction. Notably, in the parahippocampal regions, we observed a significant interaction effect between OEF and the diagnostic group (CN versus MCI) in predicting episodic memory after adjustment for multiple comparisons. Specifically for the MCI group, participants with higher parahippocampal OEF values showed higher episodic memory scores despite having an overall trend of lower OEF than the CN group in this region. Of note, this interaction effect remained significant after FDR correction only in the parahippocampal region, which is an important region in episodic memory. These findings were consistent with and without applying PVE correction, indicating that our findings are not driven by partial volume artifacts in the parahippocampal region despite its smaller size. Indeed, our parahippocampal finding aligns with the brain gray matter density signature regions associated with cross-sectional memory identified in Fletcher et al.’s work (Fletcher et al., 2024). Although statistical power in this analysis is limited, our exploratory findings underscore the importance of considering regional metabolic information in future larger studies to associate physiological imaging with cognitive impairment.

Elevated OEF in some MCI individuals may reflect compensatory mechanisms involved in cognitive reserve, which refers to the brain’s capacity to adapt in response to brain aging, damage, or diseases and maintain cognitive performance despite brain injury and associated cognitive impairment (Stern, 2012). Increased OEF may, thus, reflect the ability to compensate in the presence of pathology (Liu et al., 2016), that is, an indicator of resilience in some cases of MCI. The observed positive relationship between parahippocampal OEF and episodic memory in our MCI group aligns with recent studies of cerebrovascular reactivity (CVR). Specifically, reduced CVR in the parahippocampal gyrus was found to associate with worse memory performance in patients with MCI (Engstrom et al., 2025). CVR and OEF are generally inversely related (Nemoto et al., 2004), with higher OEF reflecting greater oxygen extraction under conditions of reduced vascular reserve; hence, this finding is consistent with our observations that higher OEF was associated with better episodic memory performance in the MCI group. These preliminary results suggest that increased OEF in regions relating to memory may represent an adaptive response that supports cognition when cerebrovascular function is compromised.

### 5.4 Limitations

The performance of the proposed Variational Bayesian inference approach for OEF mapping depends on several biophysical modeling assumptions that may affect the parameter estimation. Prior optimization was primarily performed using simulated ASE-qBOLD data at a single SNR of 70, a representative SNR of our dataset (Supplementary Fig. S1). Nonetheless, supplementary analyses indicated that the selected prior standard deviations remained consistent across different SNRs (Supplementary Fig. S2) and spanning healthy and pathological physiology. In addition, our OEF estimates were lower than those reported in previous MRI-based (He & Yablonskiy, 2007; Lu & Ge, 2008) or ^15^O-PET studies (Derdeyn et al., 2002). One contributing factor may be the overestimation of DBV, which is consistently reported in previous ASE-qBOLD studies (Stone & Blockley, 2017) and directly leads to lower calculated OEF values. Another potential source of error may be inaccuracies in R_2_^’^ estimation as diffusion effects within the tissue compartment are neglected by the static dephasing model (Yablonskiy & Haacke, 1994). Therefore, any small fluctuation in R_2_^’^ and DBV can propagate nonlinearly into OEF estimates.

Another notable limitation of the present OEF mapping approach is the absence of an inversion recovery (IR) preparation pulse to suppress the cerebrospinal fluid (CSF) signal. CSF has a long T_2_ and contributes a signal that can contaminate voxels in the brain, such as the CSF boundary, leading to abnormally elevated OEF estimates in these regions (Stone & Blockley, 2017). Future acquisitions should consider incorporating IR-based CSF suppression to improve the accuracy of OEF quantification in boundary voxels. All analyses in the present study were therefore restricted to gray matter regions of interest, which are less susceptible to this boundary artifact.

Several limitations related to the *in vivo* analysis and physiological assumptions should be considered. First, our sample size (N = 70) was moderate, with a limited number of MCI patients (N = 12), thereby limiting statistical power. Additionally, we focused only on cognitively normal and mildly cognitively impaired participants, without including people with dementia. Future research with more participants over a broader range of cognitive ability will allow us to assess whether the observed OEF alterations extend to more advanced stages of cognitive decline, and to evaluate the potential of this imaging biomarker for distinguishing between stages of neurodegeneration. Second, we assumed a uniform hematocrit level (*Hct*) of 0.40, which was selected based on values reported for the general circulation (Nicoll et al., 2012) and healthy populations (Brown et al., 1962; Jeong et al., 2021), for all participants rather than measuring it. However, *Hct* measures can vary depending on age, sex, and health conditions. Previous research suggests that anemia is linked to an increased risk of MCI, in addition to common cardiovascular risk factors (Dlugaj et al., 2016). Moreover, men typically have higher hematocrit levels than women in older populations (Hawkins et al., 1954). Variations in hematocrit and oxygen-carrying capacity could interact with physiological compensatory mechanisms, influencing overall oxygen supply. Future studies should consider hematocrit and OEF when examining metabolic adaptations in MCI and their role in compensatory responses to neurodegeneration.

Third, we did not measure CBF in this study, preventing us from validating the relationship between increased OEF and reduced CBF. Although we observed a trend of increased WMH volume and VRS in MCI compared to the CN group, we did not observe significant correlations with OEF due to the small sample population. For future studies, these relationships should be explored to understand the effects of those factors on oxygen metabolism. Fourth, the qBOLD model relies on an analytical framework that assumes deoxyhemoglobin is the primary source of magnetic susceptibility. Hence, additional susceptibility sources—such as myelin in white matter or high iron deposition in deep gray regions like the basal ganglia—will affect OEF quantification if not accounted for. Our analysis is, therefore, limited to cortical gray matter regions. Finally, there are still susceptibility artifacts occurring in inferior frontal and temporal brain areas, which could cause overestimation of OEF due to errors in R_2_^’^ estimation and limit the sensitivity of our OEF assessment in those regions (Supplementary Table S1).

## 6 Conclusions

In conclusion, this study demonstrates that the quantitative BOLD model, implemented using a Variational Bayesian framework, effectively quantifies OEF non-invasively as a tool to assess regional brain oxygen metabolism in an older cohort. The findings reveal distinct associations between OEF and cognitive performance and age, with different trends observed in cognitively normal individuals and those with mild cognitive impairment. The study highlights that OEF may reflect underlying neurophysiological changes associated with aging and cognitive impairment, offering insights into the progression of cognitive decline.

## Ethics

This study involved human participants and was reviewed and approved by the Institutional Review Board of the University of California, Davis. All subjects provided their written informed consent to participate in this study.

## Supplementary Material

Supplementary Material

## Data Availability

The datasets supporting this study can be found at the https://github.com/fanlab-ucdavis. It will be made available by the authors without undue reservation.
