# Correction to: Oxygen extraction fraction mapping between normal cognition and mild cognitive impairment in older adults using quantitative BOLD

**DOI:** 10.1162/IMAG.x.1423

**Published:** 2026-10-16

**Authors:** Linh N.N. Le, Gregory J. Wheeler, Nicholas P. Blockley, Evan Fletcher, Youngkyoo Jung, Sarah E. T. Farias, Brandon Gavett, Charles DeCarli, Audrey P. Fan

**Affiliations:** Department of Biomedical Engineering, University of California, Davis, Davis, CA, United States; School of Medicine and Health Sciences, University of Nottingham, Nottingham, United Kingdom; Department of Neurology, University of California, Davis, Davis, CA, United States; Department of Radiology, University of California, Davis, Davis, CA, United States

In the original version of this article IMAG.a.1378, due to an error in production, the abbreviation CN^a^ was incorrectly displayed in Tables 2 and 3. Specifically, CN^a^ appeared as “can” in Table 2 and “Can” in Table 3. The corrected tables are provided below.

**Table 2.** Multi-linear regression models between OEF, age, and diagnosis in the whole group (N = 70) in left and right inferior parietal regions.

	Left inferior parietal			Right inferior parietal		
Effect	β ± SE	T	P-value	β ± SE	T	P-value
Age	0.32 ± 0.12	2.60	0.012	0.33 ± 0.13	2.43	0.018
CN^a^	-0.23 ± 0.09	-2.70	0.009	-0.06 ± 0.09	-0.64	0.524
MCI^a^	0.88 ± 0.23	3.75	0.0004	0.71 ± 0.25	2.81	0.007
Region volume	-2.75 ± 10.12	-0.27	0.786	-6.93 ± 11.05	-0.63	0.533
Sex	0.36 ± 1.14	0.31	0.756	1.81 ± 1.25	1.45	0.151
Diagnosis	-39.87 ± 9.47	-4.21	<0.0001	-28.17 ± 10.33	-2.73	0.008
Age x diagnosis	0.55 ± 0.13	4.43	<0.0001	0.39 ± 0.14	2.84	0.006

In this model, OEF was the dependent variable. The independent variables are listed above.

a The analysis of the multi-linear regression model between OEF and age in each group, with diagnosis as a categorical variable.

CN: cognitively normal; MCI: mild cognitive impairment; β: standardized beta coefficient; SE: standardized error; OEF: oxygen extraction fraction.

**Table 3.** Multi-linear regression model between OEF and episodic memory in the whole group (N = 67) in left and right parahippocampal regions.

	Left parahippocampal			Right parahippocampal		
Effect	β ± SE	T	P	β ± SE	T	P
OEF	0.01 ± 0.02	0.64	0.525	0.06 ± 0.02	2.60	0.012
CN^a^	-0.04 ± 0.02	-2.89	0.005	-0.03 ± 0.02	-1.66	0.103
MCI^a^	0.07 ± 0.04	1.88	0.065	0.15 ± 0.04	3.42	0.001
Age	-0.002 ± 0.010	-0.21	0.837	-0.005 ± 0.010	-0.45	0.652
Education	0.04 ± 0.02	2.77	0.007	0.07 ± 0.02	4.14	0.0001
Region volume	-1.93 ± 10.51	-0.18	0.797	-5.11 ± 10.51	-0.49	0.629
Sex	0.70 ± 0.15	4.64	<0.0001	0.75 ± 0.15	5.15	<0.0001
Diagnosis	-1.91 ± 0.46	-4.20	<0.0001	-2.62 ± 0.54	-4.87	<0.0001
OEF x diagnosis	0.06 ± 0.02	2.82	0.007	0.09 ± 0.02	3.77	0.0004

In this model, OEF was the independent variable, while age, education, sex, regional volume and diagnosis were covariates. OEF values were corrected for partial volume effects.

a The analysis of the multi-linear regression model between episodic memory and OEF in each group, with diagnosis as a categorical variable.

CN: cognitively normal; MCI: mild cognitive impairment; β: standardized beta coefficient; SE: standardized error; OEF: oxygen extraction fraction.

