# Supplementary material for: Oxygen extraction fraction mapping between normal cognition and mild cognitive impairment in older adults using quantitative BOLD
Source: Imaging Neurosci (Camb). 2026 Sep 29;4:IMAG.a.1378. doi: 10.1162/IMAG.a.1378 (PMC13626017; doi:10.1162/IMAG.a.1378)
Supplement: Supplementary file 1 [file IMAG.a.1378_supp.pdf]

Supplementary Materials

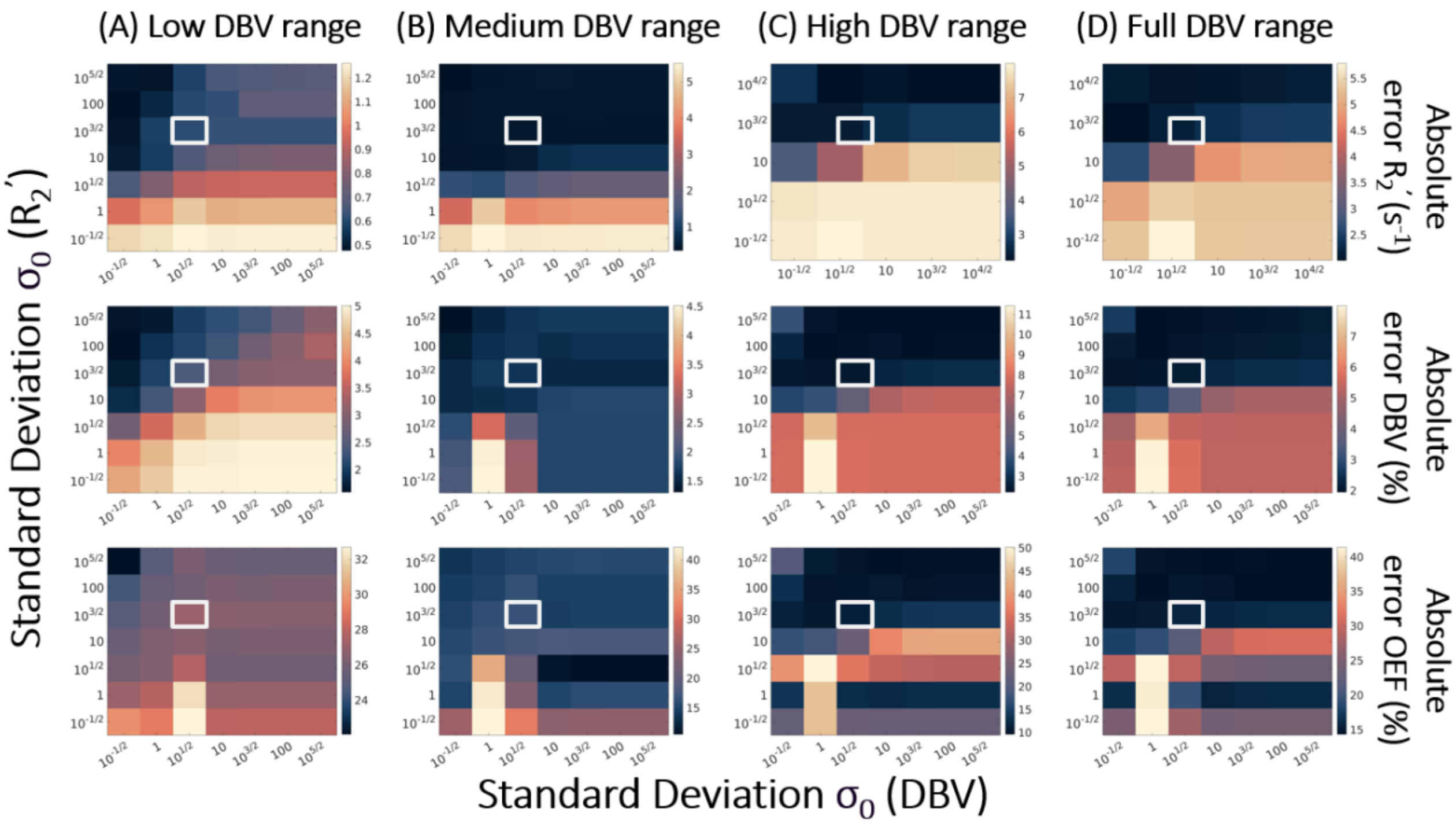

Supplementary Figure S1. Heat maps showing the effect of optimizing prior standard deviations of DBV and R_2' on parameter estimation accuracy at SNR of 70 on simulated data (as described in Section 3.2): (A) Low DBV (0.3-2.5%) range, (B) Medium DBV (2.5-7.5%) range, (C), High DBV (7.5-15%) range, and (D) Full DBV regime. The x-axis indicates the standard deviation of the DBV prior, while the y-axis indicates the standard deviation of the R_2' prior. The heat maps show absolute error on R_2' in s^{-1} (top row); DBV in % (middle row); and OEF in absolute % (bottom row). A white square indicates the optimal prior combination that produced the least absolute error across the DBV ranges (σ_0 (R_2') = $10^{3/2}$ and σ_0 (DBV) = $10^{1/2}$).

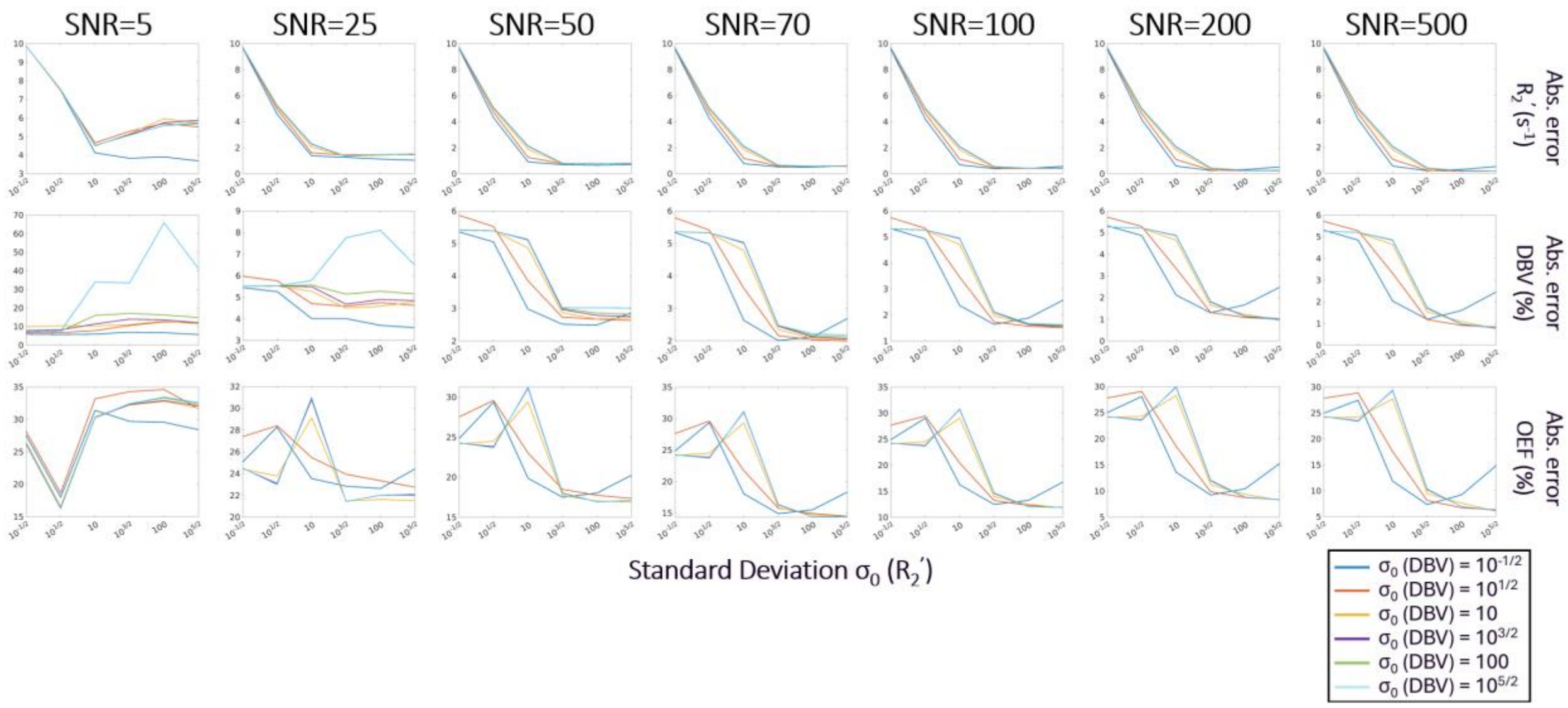

Supplementary Figure S2. Optimization of prior standard deviations σ_0 based on absolute error in parameter estimation (transverse relaxation rate (R_2' in s^{-1}), deoxygenated blood volume (DBV in %) and oxygenated blood volume (OEF in %) on simulated data at different SNR (from SNR of 5 to 500) across full DBV range. Absolute errors in OEF estimation remain non-negligible even at high SNR, particularly when compared to the regional differences (within ±4%) observed *in vivo* data. These stimulated errors reflect the mean absolute error across 1000 simulations covering a broad range of true OEF and DBV values at the voxel level. In contrast, *in vivo* OEF estimates are averaged across multiple voxels within each region of interest (ROI), which is expected to further reduce the effective noise. Therefore, this comparison highlights a limitation of the current fitting approach, which is that its sensitivity to detect subtle physiological differences in OEF measurements is constrained by the SNR of the acquisition and the choice of priors. Future work will focus on refining the prior optimization strategy to improve the sensitivity and robustness of OEF estimation and expanding the dataset to better assess the impact of noise at the group level.

Supplementary Table S1: The percent of voxels (in %) that were removed for OEF estimation in group level for OEF estimation comparing between two-tissue compartment (2C) with and without prior standard deviation optimization along with the SNR across all τ values in group level (all values are reported as mean±standard deviation)

	**2C model without prior optimization (%)**	**2C model with prior optimization (%)**	**SNR**
L caudal anterior cingulate	6.4±10.7	1.1±3.7	68.9±18.1
L caudal middle frontal	17.6±13.3	6.0±5.4	71.7±18.0
L cuneus	12.6±11.5	4.2±4.4	69.4±17.8
L entorhinal	20.4±13.0	11.2±9.5	41.8±9.9
L fusiform	20.6±10.1	11.6±4.2	46.6±10.3
L inferior parietal	21.0±12.4	5.2±4.3	74.3±17.7
L inferior temporal	37.2±15.9	28.8±7.8	40.5±10.3
L insula	9.8± 8.9	0.6±1.2	62.1±14.0
L isthmus cingulate	4.9± 5.4	0.9±1.6	44.4±9.9

L lateral occipital	22.2±12.3	8.0±5.8	67.9±17.7
L lateral orbitofrontal	39.5±17.0	28.8±8.9	47.4±11.2
L lingual	10.1± 8.8	2.5±3.1	53.2±12.9
L medial orbitofrontal	33.5±19.9	32.6±9.0	47.2±12.2
L middle temporal	24.0±12.2	10.3±4.8	56.0±12.5
L paracentral	9.1± 8.1	3.0±3.4	56.9±14.3
L parahippocampal	9.0± 7.4	4.1±4.5	41.7±9.6
L pars opercularis	9.5± 9.2	1.4±3.0	73.6±17.8
L pars orbitalis	18.5±17.5	14.0±10.4	61.2±14.2
L pars triangularis	14.8±11.7	4.0±5.7	77.2±18.6
L pericalcarine	5.6± 6.8	0.7±1.1	69.8±18.3
L postcentral	20.3±10.9	4.5±4.0	62.0±14.0
L posterior cingulate	4.6± 6.5	0.5±1.5	54.7±12.6
L precentral	20.3±11.0	4.1±3.9	66.0±15.1
L precuneus	11.9± 9.9	2.3±2.3	61.7±15.1
L rostral anterior cingulate	13.7±12.9	5.2±6.1	65.9±16.7
L rostral middle frontal	29.1±12.6	9.4±7.6	84.8±21.6
L superior frontal	35.9±11.5	9.7±5.9	87.9±24.3
L superior parietal	19.5±13.5	4.5±4.1	72.9±18.7
L superior temporal	24.2± 9.7	7.3±4.0	57.3±12.2
L supramarginal	18.2±11.9	4.2±4.0	67.3±14.3
L transverse temporal	3.9± 7.8	1.0±3.0	60.2±13.5
L hippocampus	6.6± 6.6	2.0±3.2	30.6±7.3
R caudal anterior cingulate	5.0± 7.7	1.2±3.8	69.3±17.8
R caudal middle frontal	17.9±10.4	4.6±4.4	71.9±16.9
R cuneus	12.2±11.3	3.9±3.9	69.5±18.7
R entorhinal	21.6±16.2	14.4±13	43.1±10.4
R fusiform	18.2± 9.1	11.8±3.9	43.8±10.5
R inferior parietal	19.2±10.8	4.9±4.4	72.1±16.7
R inferior temporal	35.6±14.0	24.5±6.5	45.2±11.7
R insula	10.6± 8.9	0.6±1.5	65.2±14.3
R isthmus cingulate	5.2± 6.7	0.8±1.3	41.7±9.8
R lateral occipital	21.5±12.0	8.8±6.1	64.1±17.7
R lateral orbitofrontal	40.5±17.4	32.1±9.4	48.1±11.7
R lingual	9.9± 6.5	2.4±2.2	49.2±12.5
R medial orbitofrontal	33.5±20.2	29.4±9.8	45.9±12.2
R middle temporal	23.4± 9.8	11.2±5.0	61.6±14.3
R paracentral	9.7± 8.7	3.0±3.5	56.0±13.7
R parahippocampal	8.7± 6.8	4.2±4.1	40.7±9.6
R pars opercularis	11.0± 9.3	1.3±1.7	79.0±18.6
R pars orbitalis	18.7±18.4	16.3±12.3	65.2±15.5
R pars triangularis	15.6±11.4	4.3±4.4	81.3±19.3
R pericalcarine	5.7± 6.6	0.8±1.3	66.5±18.2

R postcentral	18.7± 9.9	4.2±3.3	63.6±13.6
R posterior cingulate	4.3± 5.6	0.5±1.3	53.3±12.2
R precentral	19.7± 9.3	3.7±3.1	67.2±14.8
R precuneus	10.9± 9.4	1.8±1.7	59.7±14.4
R rostral anterior cingulate	10.6± 9.6	3.9±4.9	64.6±15.6
R rostral middle frontal	30.0±13.4	8.7±6.3	85.4±20.5
R superior frontal	34.4± 9.9	8.8±4.7	83.1±21.8
R superior parietal	18.5±10.3	4.3±3.5	72.6±18.4
R superior temporal	24.1±10.6	8.2±4.3	62.7±13.8
R supramarginal	16.9±10.5	3.9±3.6	68.8±14.6
R transverse temporal	4.2± 9.2	0.5±1.4	63.6±14.7
R hippocampus	6.3± 5.6	2.3±3.4	30.4±7.4

Supplementary Table S2. Average OEF (in %) in CN and MCI group across all participants and the absolute difference of OEF between two diagnostic groups (ΔOEF)

ROI	***P*-value**	**ΔOEF**	**CN**	**MCI**
L caudal anterior cingulate	0.991	*-0.02*	*26.19 ± 6.48*	*26.17 ± 4.68*
L caudal middle frontal	0.871	-0.27	*25.23 ± 5.02*	*24.97 ± 5.71*
L cuneus	0.032*	3.32	*23.69 ± 4.60*	*27.01 ± 5.67*
L entorhinal	0.160	-3.92	*26.56 ± 9.27*	*22.64 ± 4.76*
L fusiform	0.786	0.37	*24.68 ± 4.39*	*25.05 ± 3.63*
L inferior parietal	0.024*	3.74	*27.33 ± 4.61*	*31.07 ± 7.20*
L inferior temporal	0.704	0.41	*27.57 ± 3.18*	*27.98 ± 4.22*
L insula	0.608	-0.84	*22.67 ± 4.74*	*21.83 ± 6.84*
L isthmus cingulate	0.383	1.33	*23.48 ± 4.84*	*24.82 ± 4.53*
L lateral occipital	0.041*	3.50	*25.93 ± 5.08*	*29.44 ± 6.37*
L lateral orbitofrontal	0.925	0.15	*32.34 ± 4.98*	*32.50 ± 5.74*
L lingual	0.119	2.40	*23.30 ± 4.53*	*25.70 ± 6.00*
L medial orbitofrontal	0.569	-0.86	*27.71 ± 4.88*	*26.86 ± 3.78*
L middle temporal	0.683	0.53	*27.17 ± 3.71*	*27.71 ± 5.74*
L paracentral	0.317	1.71	*26.38 ± 5.10*	*28.09 ± 6.48*
L parahippocampal	0.103	-2.71	*24.24 ± 5.25*	*21.54 ± 4.66*
L pars opercularis	0.282	-1.61	*21.26 ± 4.50*	*19.65 ± 5.53*
L pars orbitalis	0.839	-0.46	*27.89 ± 7.02*	*27.44 ± 7.17*
L pars triangularis	0.815	0.42	*23.43 ± 5.44*	*23.84 ± 6.24*
L pericalcarine	0.030*	3.58	*18.81 ± 4.80*	*22.39 ± 6.46*
L postcentral	0.625	-0.77	*26.37 ± 4.46*	*25.59 ± 7.05*
L posterior cingulate	0.353	1.34	*25.41 ± 4.55*	*26.76 ± 4.40*
L precentral	0.449	-1.08	*25.58 ± 4.40*	*24.51 ± 4.82*
L precuneus	0.029*	3.36	*24.29 ± 4.73*	*27.65 ± 4.77*
L rostral anterior cingulate	0.702	-0.93	*26.47 ± 7.73*	*25.53 ± 7.20*

L rostral middle frontal	0.208	2.18	*25.96 ± 5.15*	*28.14 ± 6.57*
L superior frontal	0.328	1.35	*26.75 ± 4.12*	*28.11 ± 5.31*
L superior parietal	0.662	0.79	*27.50 ± 5.73*	*28.30 ± 5.52*
L superior temporal	0.855	-0.24	*26.47 ± 3.73*	*26.23 ± 5.63*
L supramarginal	0.464	1.07	*27.17 ± 4.39*	*28.23 ± 5.37*
L transverse temporal	0.412	-1.76	*24.10 ± 6.38*	*22.34 ± 8.34*
L hippocampus	0.162	-2.82	*25.92 ± 6.51*	*23.10 ± 5.01*
R caudal anterior cingulate	0.494	1.79	*27.12 ± 8.36*	*28.91 ± 7.47*
R caudal middle frontal	0.786	0.44	*23.78 ± 5.12*	*24.22 ± 4.92*
R cuneus	0.031*	3.67	*23.81 ± 4.94*	*27.48 ± 6.69*
R entorhinal	0.414	-2.20	*27.73 ± 8.72*	*25.53 ± 6.86*
R fusiform	0.925	0.10	*22.90 ± 3.51*	*23.00 ± 3.41*
R inferior parietal	0.194	2.20	*27.51 ± 4.83*	*29.72 ± 7.22*
R inferior temporal	0.890	0.12	*27.15 ± 2.83*	*27.27 ± 2.49*
R insula	0.528	0.95	*21.63 ± 4.41*	*22.59 ± 6.19*
R isthmus cingulate	0.188	1.80	*23.27 ± 4.29*	*25.07 ± 4.15*
R lateral occipital	0.113	2.72	*27.09 ± 5.01*	*29.82 ± 6.87*
R lateral orbitofrontal	0.275	1.87	*33.21 ± 5.08*	*35.08 ± 6.58*
R lingual	0.031*	3.08	*22.52 ± 4.19*	*25.60 ± 5.40*
R medial orbitofrontal	0.762	0.55	*26.91 ± 5.84*	*27.46 ± 4.85*
R middle temporal	0.331	1.13	*27.13 ± 3.65*	*28.26 ± 3.60*
R paracentral	0.101	2.98	*25.88 ± 5.45*	*28.86 ± 6.58*
R parahippocampal	0.589	-0.81	*23.37 ± 4.79*	*22.56 ± 4.23*
R pars opercularis	0.542	0.88	*20.16 ± 4.27*	*21.04 ± 5.60*
R pars orbitalis	0.387	1.93	*27.86 ± 6.87*	*29.79 ± 7.62*
R pars triangularis	0.268	2.21	*23.38 ± 6.07*	*25.59 ± 7.08*
R pericalcarine	0.010*	4.29	*18.74 ± 4.43*	*23.03 ± 7.70*
R postcentral	0.626	0.78	*25.19 ± 4.76*	*25.97 ± 6.17*
R posterior cingulate	0.223	2.05	*25.73 ± 5.36*	*27.78 ± 4.71*
R precentral	0.734	0.46	*24.31 ± 4.13*	*24.77 ± 4.88*
R precuneus	0.134	2.19	*24.01 ± 4.47*	*26.20 ± 4.98*
R rostral anterior cingulate	0.377	2.33	*26.06 ± 8.36*	*28.40 ± 7.75*
R rostral middle frontal	0.081	3.41	*25.47 ± 5.79*	*28.88 ± 7.29*
R superior frontal	0.030*	3.28	*26.04 ± 4.59*	*29.33 ± 4.98*
R superior parietal	0.458	1.27	*26.56 ± 5.48*	*27.83 ± 4.73*
R superior temporal	0.321	1.41	*25.80 ± 4.22*	*27.21 ± 5.42*
R supramarginal	0.583	0.91	*26.83 ± 4.95*	*27.74 ± 6.31*
R transverse temporal	0.900	0.24	*23.04 ± 5.29*	*23.28 ± 9.17*
R hippocampus	0.404	-1.50	*24.72 ± 5.72*	*23.22 ± 5.15*

OEF: Oxygen Extraction Fraction; CN: Cognitively normal; MCI: Mild Cognitive Impairment; L: left; R: right

***Note**: Data are in the form of mean ± standard deviation for average OEF in CN and MCI groups. The difference between two groups were compared using a multi-linear regression analysis with OEF as dependent variable and diagnosis as the independent variable. Regional brain volume was covariate. (* indicates significant P-value, $P<0.05$ before FDR correction)*

Supplementary Table S3. *Results from a multi-linear regression model examining the association between OEF and age, and diagnosis (Model 1). The table shows the interaction effect (AgexDiagnosis) in the whole group (N=70) and the effect of age for each diagnostic group: cognitively normal (CN) and mild cognitive impaired (MCI). OEF was the dependent variable, with age, sex, regional volume and diagnosis as independent variables. The table highlights regions showing a significant Age×Diagnosis interaction prior to FDR correction.*

ROI	**CN[a]**	**MCI[a]**	$Age \times Diagnosis$
L inferior parietal	T=−2.77, P=0.007*	T=3.73, P=0.0004**	T=4.43, P=<0.0001**
L precuneus	T=−1.29, P=0.200	T=1.84, P=0.069	T=2.16, P=0.034*
L superior parietal	T=−2.57, P=0.013*	T=1.64, P=0.107	T=2.41, P=0.019*
R inferior parietal	T=−0.59, P=0.557	T=2.84, P=0.006*	T=2.85, P=0.006*
R insula	T=−0.55, P=0.583	T=2.37, P=0.021*	T=2.40, P=0.019*
R isthmus cingulate	T=−0.58, P=0.563	T=2.14, P=0.036*	T=2.19, P=0.032*
R pars opercularis	T=−0.55, P=0.581	T=2.05, P=0.044*	T=2.10, P=0.039*
R precentral	T=−1.50, P=0.139	T=1.76, P=0.083	T=2.16, P=0.035*
R supramarginal	T=−0.38, P=0.703	T=2.03, P=0.046*	T=2.03, P=0.047*
R transverse temporal	T=0.38, P=0.702	T=2.72, P=0.009*	T=2.40, P=0.019*

OEF = Oxygen Extraction Fraction; L = Left; R = Right.

Note*: Values represent T-statistics and corresponding p-values from multi-linear regression models assessing the effect of age within each diagnosis group (CN and MCI) and the interaction between age and diagnosis. * indicates statistical significance at P < 0.05 (uncorrected); ** indicates significance after false discovery rate (FDR) correction.*

[a]The diagnosis as categorical variables for separate multi-linear regression models between OEF and age.

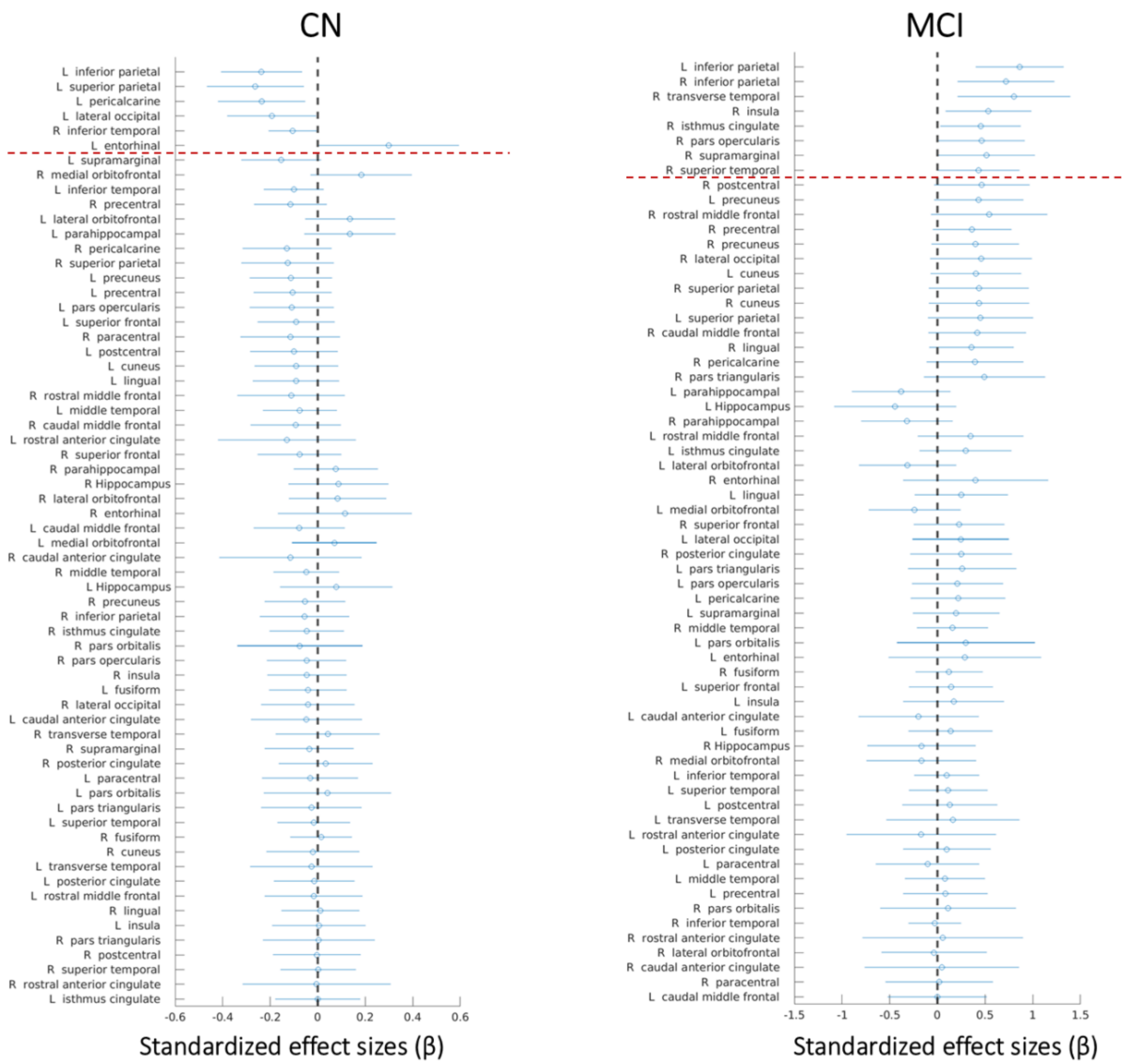

Supplementary Figure S3. Standardized effect sizes (β) and 95% confidence intervals for the association between age and OEF across brain regions, estimated from Model 1 (see Section 3.6.2). Regions are ordered by uncorrected p-values, from most to least significant (before FDR correction). The red dotted line represents the p = 0.05 threshold; effect sizes below this line correspond to non-significant associations. CN = Cognitively Normal; MCI = Mild Cognitive Impairment; L = Left; R = Right.

Supplementary Table S4. Results from multi-linear regression models examining the association between OEF and episodic memory (Model 2). The table shows the interaction effects (OEF × Diagnosis) in the whole group (N=67) and the relationship in each diagnostic group: cognitively normal (CN) and mild cognitive impaired (MCI) participants. In these

models, OEF was the independent variable, with age, education, regional volume, sex, and diagnosis included as covariates. The table reports brain regions showing a significant OEF × Diagnosis interaction prior to FDR correction.

ROI	**CN[a]**	**MCI[a]**	$OEF \times Diagnosis$
L caudal middle frontal	T=0.41, P=0.108	T=2.61, P=0.012*	T=3.05, P=0.003*
L lateral orbitofrontal	T=−0.29, P=0.133	T=1.94, P=0.058	T=2.42, P=0.019*
L middle temporal	T=0.06, P=0.197	T=2.41, P=0.019*	T=2.71, P=0.009*
L paracentral	T=−0.05, P=0.697	T=2.17, P=0.034*	T=2.08, P=0.042*
L parahippocampal	T=−0.39, P=0.005*	T=1.88, P=0.065	T=2.82, P=0.007*
L postcentral	T=0.31, P=0.089	T=2.58, P=0.012*	T=3.11, P=0.003*
L precentral	T=−1.08, P=0.130	T=2.74, P=0.008*	T=3.13, P=0.003*
L superior frontal	T=0.06, P=0.381	T=2.13, P=0.037*	T=2.28, P=0.026*
L superior temporal	T=−0.12, P=0.357	T=2.21, P=0.031*	T=2.36, P=0.022*
L supramarginal	T=−0.93, P=0.166	T=2.02, P=0.048*	T=2.43, P=0.018*
L transverse temporal	T=−1.40, P=0.062	T=1.87, P=0.067	T=2.57, P=0.013*
R caudal middle frontal	T=0.20, P=0.304	T=1.82, P=0.073	T=2.07, P=0.043*
R paracentral	T=−0.35, P=0.795	T=2.58, P=0.012*	T=2.38, P=0.021*
R parahippocampal	T=−0.26, P=0.109	T=3.44, P=0.001*	T=3.77, P<0.0001**
R pars opercularis	T=−1.63, P=0.085	T=1.98, P=0.052	T=2.58, P=0.012*
R postcentral	T=−0.36, P=0.197	T=2.24, P=0.029*	T=2.61, P=0.012*
R rostral middle frontal	T=−0.43, P=0.111	T=1.98, P=0.053	T=2.51, P=0.015*
R superior frontal	T=−1.62, P=0.200	T=1.89, P=0.063	T=2.26, P=0.027*

OEF: Oxygen Extraction Fraction; CN: Cognitively normal; MCI: Mild Cognitive Impairment; L: left; R: right

***Note**: Values represent T-statistics and P-values from multi-linear regression models assessing the association between OEF and episodic memory within each diagnosis group, as well as the interaction between diagnosis groups in the whole group. * indicates P < 0.05 (uncorrected); ** indicates significance after false discovery rate (FDR) correction.*

[a]The diagnosis as categorical variables for separate multi-linear regression models between OEF and episodic memory cognitive scores.

Supplementary Method:

1. Bayesian inference for prior selections

Variational Bayesian inference is an approach to infer the conditional distribution over the laten variables given the observations. In our case, we aim to infer the parameters R_2' (the reversible transverse relaxation rate) and DBV (deoxygenated blood volume) given observed ASE (asymmetric spin echo) measurements. The ASE signal is modeled as a function of these parameters, incorporating prior knowledge and noise characteristics. Using the variational approach, we approximate the true posterior distribution $P(R_2', DBV|ASE\ data)$ with a tractable distribution $q(R_2', DBV)$, optimizing it by minimizing the Kullback-Leibler (KL) divergence between q and the true posterior. This allows for efficient inference to iteratively refine the estimates of R_2' and DBV. The

Variational Bayes (VB) method provides an efficient way to optimize and select priors, including those for standard deviations σ_0 (Chappell et al., 2009). By approximating the posterior distribution with a simpler form, variational Bayes enables the estimation of hyperparameters such as standard deviations while balancing computational efficiency and model accuracy. This method is especially valuable in complex models where traditional inference techniques may be computationally intensive.

2. Vascular risk score

The ADRC vascular risk score (VRS) was calculated based on five factors: hypertension, diabetes, hyperlipidemia, coronary artery disease, and cerebrovascular disease (DeCarli et al., 2019). Each factor was scored as 1 if present and 0 if absent, with a maximum score of 5. To handle missing data, the categorical score was converted into a continuous percentage (0-1) by averaging non-missing values. The VRS, recently developed for consistency across studies, was derived from participants' medical histories, records, and medications reported at the initial clinic evaluation.

3. White matter hyperintensities segmentation

White matter hyperintensities (WMH) were segmented using T_1 and FLAIR MRI images through a semiautomated method utilizing in-house-built computer algorithms and previously described in another study (DeCarli et al., 2005; Maillard et al., 2022, 2024). In summary, the technique involves detecting voxels in the FLAIR image with intensities greater than 2.5 standard deviations above the normalized mean, ensuring the intensity mode is standardized. WMH probability priors were also applied in the algorithm from a predefined atlas to native images to more accurately reflect the likelihood of WMH occurrences.
